# Network Models of TEM β-Lactamase Mutations Coevolving under Antibiotic Selection Show Modular Structure and Anticipate Evolutionary Trajectories

**DOI:** 10.1371/journal.pcbi.1002184

**Published:** 2011-09-22

**Authors:** Violeta Beleva Guthrie, Jennifer Allen, Manel Camps, Rachel Karchin

**Affiliations:** 1 Department of Biomedical Engineering and Institute for Computational Medicine, Johns Hopkins University, Baltimore, Maryland, United States of America; 2 Department of Environmental Toxicology, University of California Santa Cruz, Santa Cruz, California, United States of America; University of Texas at Austin, United States of America

## Abstract

Understanding how novel functions evolve (genetic adaptation) is a critical goal of evolutionary biology. Among asexual organisms, genetic adaptation involves multiple mutations that frequently interact in a non-linear fashion (epistasis). Non-linear interactions pose a formidable challenge for the computational prediction of mutation effects. Here we use the recent evolution of β-lactamase under antibiotic selection as a model for genetic adaptation. We build a network of coevolving residues (possible functional interactions), in which nodes are mutant residue positions and links represent two positions found mutated together in the same sequence. Most often these pairs occur in the setting of more complex mutants. Focusing on extended-spectrum resistant sequences, we use network-theoretical tools to identify triple mutant trajectories of likely special significance for adaptation. We extrapolate evolutionary paths (n = 3) that increase resistance and that are longer than the units used to build the network (n = 2). These paths consist of a limited number of residue positions and are enriched for known triple mutant combinations that increase cefotaxime resistance. We find that the pairs of residues used to build the network frequently decrease resistance compared to their corresponding singlets. This is a surprising result, given that their coevolution suggests a selective advantage. Thus, β-lactamase adaptation is highly epistatic. Our method can identify triplets that increase resistance despite the underlying rugged fitness landscape and has the unique ability to make predictions by placing each mutant residue position in its functional context. Our approach requires only sequence information, sufficient genetic diversity, and discrete selective pressures. Thus, it can be used to analyze recent evolutionary events, where coevolution analysis methods that use phylogeny or statistical coupling are not possible. Improving our ability to assess evolutionary trajectories will help predict the evolution of clinically relevant genes and aid in protein design.

## Author Summary

Understanding how new biological activities evolve on the molecular level has critical implications for biotechnology and for human health. Here we collect a database of mutations that contribute to the evolution of β-lactamase resistance to inhibitors and to new β-lactam antibiotics in bacterial pathogens, such as *Escherichia coli*. We compiled a database of TEM β-lactamase sequences evolved under antibiotic pressure and identified functional interactions between individual residue positions. We visualized these complex molecular interactions as a network and used network theory to derive information regarding the origin of individual mutations and their contribution to the observed resistance. Our approach should help interpret sequence databases for clinically relevant proteins undergoing high mutation rates and under selective (drug, immune) pressure, such as surface proteins of pathogens (particularly of RNA viruses such as HIV) or targets for chemotherapy in microbial pathogen or tumor cells. Notably, our approach only requires sequence data; detailed phylogenetic or tertiary structure information for the target gene is not necessary. Our analysis of how individual mutations work together to produce new biological activities should help anticipate evolution driven by a variety of clinically-relevant selections such as drug resistance, virulence, and immunity.

## Introduction

Evolutionary biology seeks to understand how proteins rapidly evolve novel functions and adapt to new environments, while retaining their functional specificity [1]–[4]. Improved understanding of the genetic basis of adaptive evolution should help anticipate the functional impact of mutations, which has critical clinical and biotechnological implications [2], [3], .

It has been noted that for a given protein target under selective pressure, the contribution of individual amino acid substitutions to adaptation is highly variable [1], [4]. A few residues have a large impact on increasing fitness under selective conditions, whereas the contribution of most residues is more modest [6]. The difference between these two classes of mutations cannot always be explained only by properties of the specific sites: The impact of mutations is context-dependent and reflects a complex network of interactions between multiple residues within a protein [1], [4], [7].

Bacterial β-lactamases, enzymes that break up the functional ring of β-lactam antibiotics, are a good model system for the study of genetic adaptation. The reason is that acquisition of resistance to inhibitors and newer β-lactam antibiotics [7], [8] requires only a small number of mutations. This is also a system where the impacts of individual mutations on adaptive fitness can be readily assessed.

Since the discovery of a β-lactamase known as TEM-1 in 1963, over 170 mutants have been identified in clinical environments, in addition to dozens more described in laboratory evolution experiments (reviewed in [9]). Here we compiled a comprehensive database of clinically or experimentally derived TEM-1 β-lactamase mutant sequences. Our assumption is that the majority of mutations within these mutants involve a degree of positive selection, and that coevolution of two given residue positions points to a functional interaction between them. We generated a graphic representation of these genetic interactions — an undirected network of mutated residue positions, in which edges are weighted based on co-occurrence frequency relative to the frequency of the constituent single mutations. We found that this network segregates mutant positions according to known selective pressures, namely broad-spectrum, extended-spectrum and inhibitor resistance.

We then focused on a network model of mutant positions involved in extended-spectrum resistance, which is the best-represented resistance phenotype class in our TEM mutant sequence database. We reasoned that generating adaptive evolutionary trajectories involves assembling combinations of mutations that fulfill the specific functional milestones required for genetic adaptation. If we assume that every mutant position represents a potential functional milestone, adaptation involves information transfer across the network. We focused on the most experimentally tractable evolutionary trajectories (trajectories involving three mutations) and identified mutation paths that facilitate the transfer of information across the network as paths of likely special significance for adaptation. The particular significance of these evolutionary trajectories identified by our analysis is demonstrated because they frequently increase protection over constituent double mutation pairs. Even though most of these trajectories had been previously described, our ability to identify them implies that our analysis has predictive value because it had no information about the original sequence context of the co-occurring pairs of mutations.

Our network approach attempts to maximize the amount of genetic information that can be derived from sequences, in the setting of rapid evolution under defined selective pressures, such as drug resistance, virulence, or immune evasion. Detailed phylogenetic or structural information is not required for our method in its current form, but our approach is amenable to the incorporation of biophysical, tertiary structure, and phylogeny variables.

## Results

In order to study how new biochemical activities arise during evolution, we compiled a database of TEM mutant sequences that have evolved under antibiotic selective pressure. Our database includes clinical (n = 144 [10], [11]) and laboratory evolved (n = 217 [12]–[26]) sequences. This database of TEM β-lactamase mutants is available in Tables S1 and S2 (annotated amino acid residue substitutions) and in Datasets S1-S8 (FASTA sequences and references).

Mutations within TEM β-lactamase sequences can be grouped into the following three phenotypic classes, corresponding to specific functional selections: mutations associated with resistance to penicillins and some earlier generation cephalosporins (broad-spectrum resistance or Class 2b), mutations conferring resistance to later generations of cephalosporins and monobactams (extended-spectrum resistance or Class 2be), and mutations that make β-lactamases resistant to inhibitors (inhibitor resistance or Class 2br) [27].

Our first assumption was that a majority of mutations present in our database would have undergone a degree of positive selection. This assumption was based on the fact that the rapid evolution of β-lactamases in recent years has been linked with the widespread use of antibiotics [28], [29]. Also, a concordance between clinical and experimental β-lactamase evolution has been established [9]. PAML (codeml) analysis [30] of the naturally occurring sequences, further supports our assumption by showing enrichment of non-synonymous vs. synonymous mutations (ω>1) in most residue positions (Table S3).

**Figure 1:**
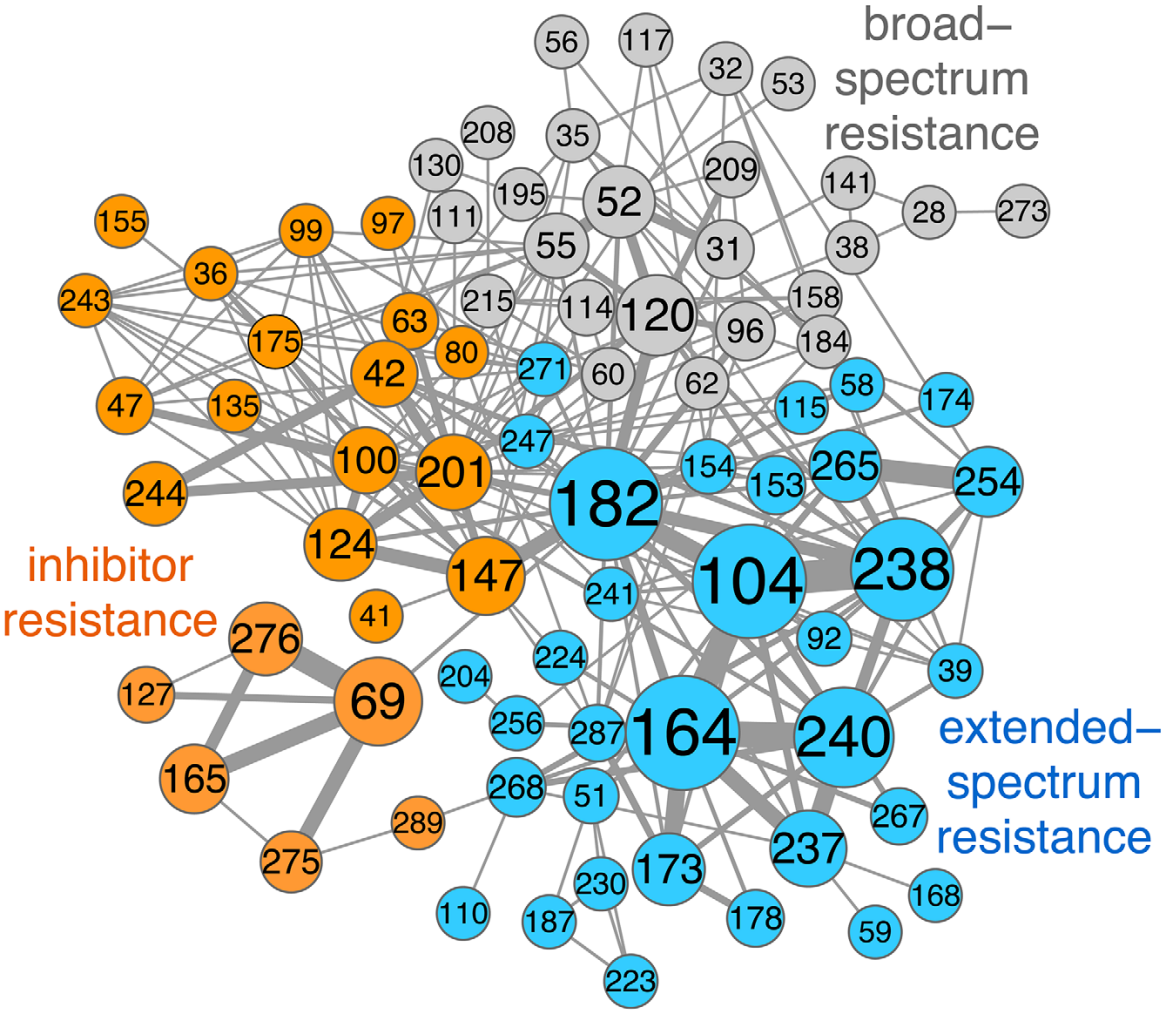
The TEM coevolution network and its communities. The network was constructed based on frequencies of co-occurring mutated residue positions in 363 mutant TEM β-lactamase sequences. Node size is proportional to how well connected a node is to its neighbors and how many neighbors it has (weighted degree centrality, Methods). Link thickness is proportional to the number of sequences in our database in which both positions are mutated, normalized by the number of sequences in which only one or the other position is mutated (Methods). Node (residue) numbers are shown in Ambler notation. The Clauset community-finding algorithm [31] identified three major communities, corresponding to three Bush-Jacobi β-lactamase phenotype classes: broad-spectrum antibiotic resistance or 2b (gray), extended-spectrum antibiotic resistance or 2be (blue) and inhibitor resistance or 2br (orange). Mutated positions with phenotypic effects documented in [9]: extended-spectrum resistance 51, 173, 237, 240, 39, 164, 104, 238, 153, 265, 92, 224; inhibitor resistance 165, 69, 275, 276, 244, 201; inhibitor and extended-spectrum resistance: 182, 268. Image created with CytoScape [78].

**Figure 2:**
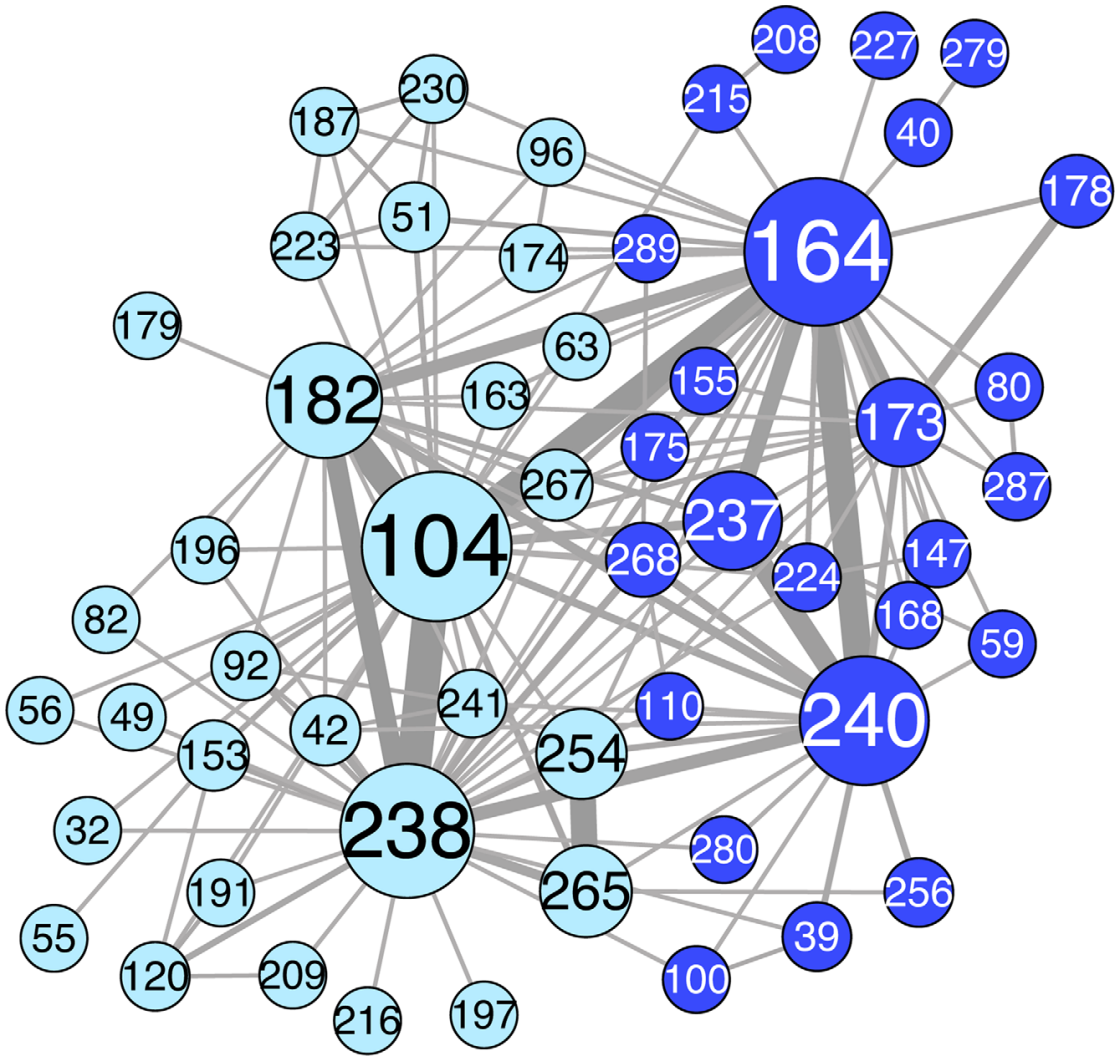
The TEM extended-spectrum community network and its two subcommunities. The network was constructed in the same way as Figure 1, but here we only used sequences associated with extended-spectrum antibiotic resistance. We identified two large subcommunities, the first containing the active-site residue 238 (light-blue), and the second containing the active-site residue 164 (dark-blue). Node size is proportional to how well connected a node is to its neighbors and how many neighbors it has (weighted degree centrality, Methods). Link thickness indicates how frequently two residues (nodes) are mutated in the same sequence, normalized by the number of sequences in which only one or the other position is mutated (Methods). Image created with CytoScape [78].

Our second hypothesis was that co-occurrence of pairs of mutated residue positions within the same sequence is indicative of a functional relationship between these positions. We constructed an undirected, weighted network representation of co-occurring residue pairs in order to map out potential functional interactions underlying the evolution of β-lactamase under antibiotic selective pressure. In this network model (shown in Figures 1 and 2), mutated residue positions are represented as nodes. Links connect pairs of nodes corresponding to residue pairs observed to be co-mutated in at least one TEM sequence. In order to give an idea of how important each residue node is for the network, node size is proportional to weighted degree centrality, which shows how well a node is connected to its neighbors and how many neighbors it has (Methods). To indicate the potential strength of the interaction, links within our network are weighted in proportion to the number of residue pair co-occurrence events. Epistatic information is implicitly incorporated into link weights, through a normalization factor comparing the frequency of a given mutated position pair with the frequency of the corresponding individual mutations at the two positions (Methods).

### The TEM coevolution network is scale-free and modular

The weighted degree distribution of the network, *i.e.* the aggregate weight of the links incident on each individual node, reveals overall few highly connected nodes, with a majority of nodes exhibiting low connectivity (Figure S1).

The TEM coevolution network also has a modular structure, with a modularity score [31] Q = 0.522, where 0≤Q≤1.0; This modularity occurs at two levels: at a broad (community) level and at a narrower (subcommunity) level (Figures 1 and 2). The Clauset community-finding algorithm [31] (Methods) identified three major network communities (Figure 1). We found a clear correspondence between each of these communities and each of the β-lactamase phenotype classes defined by Bush and Jacobi [27]: 1) broad-spectrum antibiotic, 2) extended-spectrum antibiotic, and 3) inhibitor resistance. The broad-spectrum antibiotic community includes mutations previously reported as nearly neutral or as preserving the parental TEM-1 phenotype, since catalytic efficiency for broad-spectrum β-lactams has evolved to “perfection” in TEM-1 [32]. The extended-spectrum community contains mutations at eight positions that are known to extend the substrate spectrum of the enzyme: 39, 51, 104, 164, 173, 237, 238, 240 [9], [17], [19], [21], [24], [33]–[41], as well as four stabilizing mutations: 153, 182, 224, 268 [9], [25], [39], [41]–[43]. Likewise, the inhibitor community contains five positions known to confer inhibitor resistance: 69, 165, 244, 275, 276 [9], [13], [44]–[49] and three enhancer stabilizing mutations: 147, 201, 275 [9], [25], [43], [45], [50]–[52].

On a narrower level, within the two adaptive community networks (the extended-spectrum and inhibitor-resistant community networks), we found subcommunities, *i.e.* subnetworks of densely connected nodes. These subcommunities likely represent parallel strategies of adaptation within a community's phenotype class, namely trajectories leading to different local maxima within the fitness landscape (Discussion).

### Functional information within the 2be community network

We reasoned that by analyzing the connectivity of the TEM β-lactamase coevolution network, we could extract functional information about amino acid residue positions in this enzyme. We focused our analysis on the extended-spectrum community, which is the adaptive community network based on the largest number of available mutant sequences.

We used the occurrence count of mutations at a given position as an indication of functional importance for extended-spectrum β-lactamase resistance (Table 1, column 2) and compared these counts with two well-established network centrality metrics: the degree and the node betweenness centrality ranks (Table 1, columns 3, 4). The degree centrality rank is an indication of how well connected a node is to its neighbors and how many neighbors it has (Methods). Node betweenness centrality can be interpreted as a measure of information flow through a given node from the entire community. All the frequent mutant positions (n>10) ranked high by both metrics, suggesting that node centralities in the network are good indicators of the corresponding residue functional relevance for extended-spectrum β-lactamase resistance. However, the sensitivity of the metrics is revealed in the less frequently mutated positions such as 120, 51, and 268, as these would not have been predicted to have a high functional impact based on frequency alone. Within this category, node betweenness centrality ranks tend to be higher than node degree ranks, suggesting that node betweenness centrality is a more sensitive metric for assessing the functionality of individual nodes within the network.

**Table 1:** The mutated residue positions most important for TEM extended-spectrum antibiotic resistance, according to measures from network theory (centrality rankings).

Residue Number*	Count within Data- base	Node Degree Rank	Node Between-ness Rank	Described Function	References
104	48	1	1	The long K chain of E104K mutants interacts directly with carboxylic acid group of the substrate.	[9], [73]
164	48	2	2	Forms two salt bridges, to E171 and D179, critical for correct positioning of E166. The smaller mutant chain collapses the Ω-loop, resulting in an active site with greater accessibility.	[9], [74]
238	38	3	3	Expands the active site either by repositioning the B3 β-strand or by tilting the Ω-loop	[73], [75]
240	31	4	4	Interacts with substrate; possibly stabilizing.	[73], [76]
182	27	5	5	Increases the thermodynamic stability of the protein; could suppress misfolding and aggregation caused by other mutations. Acts as a global suppressor.	[9], [43], [77]
265	20	7	9	Unknown mechanism. Possibly important for enzyme stability.	[9]
237	9	6	8	Introduces another H-bond with carbonyl group of β-lactam ring.	[9], [73]
173	5	9	6	Increased resistance, specific for subset of cephalosporins.	[9]
120	3	17	8	Unknown mechanism. Possibly important for enzyme stability.	[25], [50], [51]
254	3	8	N/A	Unknown mechanism. Possibly stabilizing.	[9], [25]
51	2	15	7	Unknown mechanism. Possibly important for both enzyme activity and stability.	[9], [35]
268	2	10	8	Unknown mechanism. Possibly stabilizing.	[9]

Degree centrality rank is based on how well connected a node is to its neighbors and how many neighbors it has (Methods). We interpret betweenness centrality as a representation of the information flow through a node from the entire community (Methods).

*Based on Ambler TEM β-lactamase numbering scheme [65]. Mutated residues that are highly ranked by the network centrality metrics have known functional impact previously described in the literature. While many of the mutations known to contribute to extended-spectrum resistance are highly frequent, the network also ranks highly the less frequent mutations with known contributions.

### Using the network to identify evolutionary trajectories of potential special significance for adaptation

Each link in the TEM coevolution network represents a potential step within an adaptive evolutionary trajectory. Although, by construction, all two-node paths have been seen in natural or laboratory evolution, by defining longer paths within the network, we should be able to derive evolutionary trajectories consisting of more than two mutations. We chose to analyze two-edge (three-node) shortest paths, each of which represents an evolutionary trajectory that produces a triple mutant sequence, because they are the most tractable to enumerate and explore.

Our hypothesis was that adaptive evolution often involves discrete steps in the form of functional modifications: improved active site fit to a new substrate, suitable chemical environment in the active site, increased thermodynamic stability, *etc.* Therefore, adaptive evolutionary trajectories can be conceptualized as a successful combination of functional milestones. In this scenario, the evolution of new biochemical activities involves transfer of information within our network, where each node is a potential functional milestone. We reasoned that efficient information transfer would improve the chances of generating mutant combinations with high fitness.

We identified evolutionary trajectories of special significance for adaptive evolution based on shortest path betweenness-centrality — a metric that can be interpreted to measure the efficiency of information transfer through the network. We found that a subset of all possible three-node paths in the network (48 out of 214) had a shortest path betweenness centrality greater than zero. These triple mutant trajectories are listed in Table 2, ranked in descending order of betweenness centrality. Shown is also the number of times (count) that each residue position in the trajectory was seen mutated in the 201 extended-spectrum resistant TEM sequences in our database. Note that many nonzero betweenness trajectories consist of at least one infrequent mutation and therefore would not have been predicted as critical based on frequency alone. Note also that these 48 triplets consist of combinations of only 16 residue positions out of a total of 55 residue positions in the network. These positions could be of special significance for the evolution of extended-spectrum β-lactamase resistance (see below).

**Table 2:** Prediction of critical triple mutant evolutionary trajectories in the extended-spectrum antibiotic resistance community.

Evolutionary Trajectory	BetweennessCentrality	Count	Previously Reported in Clinical and/or Laboratory-evolved Isolates
238_104_164	96	48,48,38	TEM-008*,TEM-134*, [36] ***
173_164_104	92	48,48,5	[17] **
182_104_164	66	27,48,48	TEM-043*,TEM-063*, [36] ***
240_164_104	62	31,48,48	TEM-046*
268_240_164	41	2,31,48	TEM-136*, [36] ***
120_238_104	39	3,38,48	[24] **, [36] ***
39_240_164	32	1,31,48	[36] ***
237_164_104	28	9,48,48	TEM-130*, [36] ***
104_238_153	23	48,38,9	TEM-021*, [36] ***
240_164_173	22	31,48,5	TEM-132*, [36] ***
104_164_40	18	48,48,1	
238_104_51	16	38,48,2	
215_104_164	15	48,38,20	TEML-136*
104_238_265	15	2,48,48	[17] **, [15] **, [36] ***
39_240_238	12	1,31,38	
182_104_51	11	27,48,2	
173_164_51	9	5,48,2	
215_104_238	8	2,48,38	
182_238_120	7	27,38,3	[24] **
240_164_51	6	31,48,2	
224_164_173	6	3,48,5	[19] **
173_164_237	6	5,48,9	[17] **, [29] ***
224_164_240	5	3,48,31	
173_164_40	4	27,38,20	
182_104_215	4	27,48,2	
182_238_153	4	5,48,1	[36] ***
240_238_153	4	31,38,9	
182_238_265	4	27,38,9	[36] ***
51_164_40	3	20,38,31	
40_164_240	3	2,31,9	
224_164_251	3	3,48,2	
51_164_237	3	1,48,31	
268_240_237	3	2,48,1	TEM-136*, [36] ***
265_238_240	3	2,48,9	[36] ***
39_240_237	2	3,38,9	[36] ***
39_240_268	2	2,38,3	
120_238_153	2	31,38,3	[24] **
240_238_120	2	3,48,9	
120_238_265	2	1,31,2	
268_238_120	2	2,38,9	
268_238_153	2	3,38,20	
224_164_237	2	1,31,9	[36] ***
224_164_40	1	2,38,20	
237_164_40	1	9,48,1	
51_104_215	1	48,48,3	
104_164_224	1	20,38,9	[36] ***
265_238_153	1	3,48,1	
268_238_265	1	2,48,2	

Triple mutant trajectories are shown as an ordered list of three residue positions, where an ordered pair represents a link in the network. The shortest path betweenness centrality is listed for each triple mutant trajectory, in descending order. We interpret the betweenness centrality of a trajectory as a representation of information flow through this path for the entire community network: Trajectories with high betweenness centrality have the highest information flow (Methods). The count shows the number of times that each residue position in the trajectory was seen mutated in the 201 extended-spectrum resistant TEM sequences in our database. Note that many trajectories consist of at least one infrequent mutation and therefore would not have been predicted as critical based on frequency alone. Some of the triple mutants have been seen either alone or in combination with other mutations in clinical isolates (*), in laboratory-evolved isolates that were included in our database (**), or in laboratory-evolved isolates that were not in our network database, from a recent report that was published after our analysis was completed (***) [36].

### Evidence that trajectories with high betweenness centrality are significant for adaptation

We investigated the significance of betweenness centrality as an indicator of potential adaptive evolution. Below we show that: 1) the triple mutant trajectories listed in Table 2 as of potential special significance for adaptation are enriched for triple mutants that have been previously reported; 2) the reported triple mutant combinations consistently increase extended-spectrum resistance over constituent double mutants, confirming they resulted from a functional selection; 3) using reported triplet mutants as a proxy for increased resistance, we can estimate the “success rate” of our method. Our success rate is considerably higher than what would be anticipated based on the simple assumption that the most successful triplet combinations consist of the most frequent single mutations in our database. Together, these three lines of evidence strongly support the predictive value of our extrapolation to triple mutant evolutionary trajectories.

1. *Nonzero betweenness centrality triplets frequently identify triple mutants associated with extended-spectrum resistance.* In addition to listing nonzero shortest path betweenness centrality trajectories, Table 2 also shows which of these trajectories were previously reported in clinical or experimental studies. Trajectories are listed in descending order of betweenness centrality value. We noted that this list is rich in triple mutant combinations that have been previously described in clinical or experimental reports, with 23 previously described out of the 48 predicted paths. In addition, we found a strong association between the chance of having been previously reported and the corresponding shortest path betweenness centrality value: while all of 10 top-ranked triplet paths are already known; only 1 of the 6 paths with the lowest positive betweenness centrality (value of 1) is known.
2. *Trajectories produced during experimental or natural evolution increase extended-spectrum resistance.* We interpreted the occurrence of a given path (evolutionary trajectory) in clinical isolates or published laboratory evolution experiments as an indication of likely fitness advantage, *i.e.* of likely increased resistance to extended-spectrum β-lactam antibiotics. We confirmed this interpretation experimentally, using cefotaxime as a representative extended-spectrum β-lactam antibiotic as done previously in similar studies [7], [25], [36]. We tested 15 triple mutants that span a range of shortest path betweenness centrality values, by measuring growth (in centimeters) along an LB agar plate containing a cefotaxime gradient (Methods). Of these 15 triple mutant trajectories, 9 had already been described, and 6 were new. The results (Table 3) show that observed mutants consistently increased resistance over both ordered, constitutive pairs: 8 out of the 9 previously reported triple mutants. By contrast, none of the non-observed mutant sequences we tested improved on both constitutive double mutants. These results confirm the intuitive notion that combinations of mutants that increase fitness are more likely to have been selected during evolution of TEM β-lactamase under extended-spectrum antibiotic selection and therefore reported.
3. *The success rate of our analysis is not due to chance.* Our experimental results show that observed triple mutants consistently increase cefotaxime resistance. Thus, we reasoned that to be reported as having extended-spectrum resistance is a viable proxy for having increased fitness. By this logic, the predictive success rate of our method is 23 out of 48. To demonstrate that this success rate is not due to chance, we ran a simulation in which we randomly selected 48 triple mutants only from TEM residue positions previously reported in association with extended-spectrum antibiotic resistance. We sampled these positions according to their mutation frequency in our database. The 10,000 random sets of 48 triple mutants selected in this way followed a normal distribution, as expected. This simulation produced an average success rate of 12.8±3.08 observed triplets out of 48. Since our success rate of 23 out of 48 is well outside the range of standard error, our analysis has predictive value (Discussion).

We next addressed the functional significance of links present in our network. To that end, we compared the level of resistance of pairs of mutations present in nonzero betweenness centrality trajectories to their constituent mutations (Table 4; original measurements in Table S4). Given that our network is largely constructed with mutations that have experienced some degree of positive selection, and that mutant positions are linked when they co-occur in the same sequence, we expected a predominance of positive interactions. To our surprise, we found that almost half of the 21 pairs tested showed a negative trend, and that 5 out of 12 significant sign-epistatic interactions were negative. When we compared the effect of single mutations on mutation pairs in triple mutant trajectories (Table 5), we found 8 significant negative epistatic interactions versus 19 positive ones. Overall, our analysis revealed a surprising number of negative interactions: 22 out of 60 tested interactions had a negative trend, which was statistically significant in 13 cases. Thus, while links in our network represent potential functional interactions, these links are not necessarily indicative of positive epistasis. In fact, their interaction is frequently negative. Because all the pairs we tested co-occurred in at least one TEM sequence, we inferred that the interaction was positive in the original sequence, *i.e.* in the presence of additional mutations.

**Table 3:** Triple mutant trajectories critical to the extended-spectrum antibiotic community network are shown experimentally to increase resistance over their constituent doublets.

Triplet	Between ness Centrality	Reported?	Resistance outcome [cm]	Doublet 1	Resistance Outcome [cm]	Triplet Improvement over Doublet 1 [cm]	Doublet 2	Resistance Outcome [cm]	Triplet Improvement over Doublet 2 [cm]
104_164_173	92	Y	16.49	104_164	8.42	8.07	164_173	6.95	9.54
182_104_164	66	Y	16.86	182_104	2.82	14.04	104_164	8.42	8.44
39_240_164*	32	Y	9.10	39_240	2.16	6.94	240_164	9.48	-0.38**
104_238_153	23	Y	17.65	104_238	16.83	0.82**	238_153	11.5	6.15
240_164_173	22	Y	17.48	240_164	9.48	8.00	164_173	6.95	10.53
104_164_40	18	N	5.06	104_164	8.42	-3.36	164_40	2.13	2.93
238_104_51	16	N	1.88	238_104	16.83	-14.95	104_51	1.65	0.23**
104_238_265	15	Y	19.40	104_238	16.83	2.57	238_265	10.84	8.56
39_240_238	12	N	9.59	39_240	2.16	7.43	240_238	12.04	-2.45
182_104_51	11	N	2.54	182_104	2.82	-0.28**	104_51	1.65	0.89
173_164_51	9	N	1.79	173_164	6.95	-5.16	164_51	1.9	-0.11**
215_104_238	8	N	11.26	215_104	2.39	8.87	104_238	16.83	-5.57
182_238_153*	4	Y	17.95	182_238	16.17	1.78	238_153	11.5	6.45
120_238_153	2	Y	14.36	120_238	7.22	7.14	238_153	11.5	2.86
104_164_224*	1	Y	9.31	104_164	8.42	0.89***	164_224	3.9	5.41

**Figure 3:**
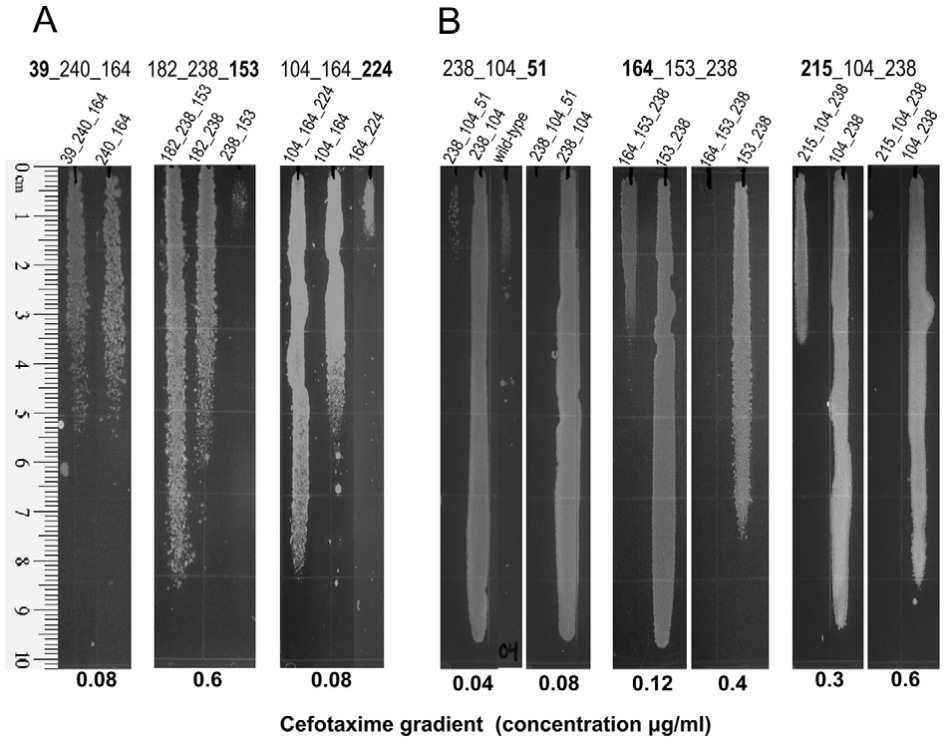
Cefotaxime plate growth assays for selected clones. Cultures of cells expressing the β-lactamase mutants listed at the top of the gradients were stamped on LB plates containing a cefotaxime gradient. The direction of the gradient is from top (minimal concentration) to bottom (maximal concentration). The maximal concentration of the gradient is listed at the bottom. Note that in part B more than one concentration is shown to cover the wide range of resistance phenotypes of the panel of mutants being tested. (A) Two mutant triplets predicted to be of special significance by our analysis but that were not present in the sequence database used to build the network but were subsequently reported in [36], and a third triplet also predicted by our analysis but that showed only a marginal increase. Only the doublet with the highest level of resistance is shown. (B) Triplets with the strongest negative epistatic effects. The mutation responsible for the negative effect is highlighted in bold.

Each mutant trajectory is shown as an ordered list of three mutated residue positions (column 1). Each ordered pair of mutated residue positions represents a link in the extended-spectrum community network. The shortest path betweenness centrality is listed for each trajectory (column 2). This metric is unitless and is a measurement of the path's importance in the network. 9 of the 15 tested trajectories were reported in clinical or directed evolution isolates (column 3). The level of cefotaxime resistance (an indicator of extended-spectrum antibiotic resistance) is shown in centimeters of linear growth on a 0.04 µg/ml cefotaxime gradient. The level of resistance is shown for each triple mutant trajectory (columns 1 and 4) and its two ordered constituent double mutants (columns 5 and 6, and 8 and 9). The differences representing the improvement in resistance conferred by the triple mutant trajectory with respect to each double mutant, is shown in columns 7 and 10. Trajectories marked with * had not been reported when this work was done and were not included in input to the network. They were subsequently reported in a recent publication [36]. (**) Triplet improvement over pair is outside the margin of standard error (for the number of replicates (n) refer to Table S4). (***) Improvement is outside the margin of standard error if the variability between gels is subtracted out (Figure 3).

**Table 4:** Experimentally determined epistatic interactions between single mutations in the extended-spectrum antibiotic resistance community network.

M1	M1 Growth [cm]	M2	M2 Growth [cm]	M1_M2 Growth [cm]	M1_M2 - (M1+M2) [cm]	Significant Epistastic Effect*
Q39R	2.09	G238S	9.61	7.76	-2.35	
Q39R	2.09	E240K	1.58	2.16	0.08	
L40W	2.08	R164H	3.43	2.13	-1.79	negative
L51P	1.93	E104K	2.20	1.65	-0.89	negative
L51P	1.93	R164H	3.43	1.90	-1.87	negative
E104K	2.20	H153R	2.17	2.73	-0.05	
**E104K**	**2.20**	**R164H**	**3.43**	**8.42**	**4.38**	**positive**
E104K	2.20	I173V	2.10	10.84	8.13	positive
**E104K**	**2.20**	**M182T**	**2.15**	**2.82**	**0.06**	
E104K	2.20	K215E	1.90	2.39	-0.12	
E104K	2.20	A224V	1.86	1.90	-0.57	
**E104K**	**2.20**	**G238S**	**9.61**	**16.83**	**6.61**	**positive**
R120S	1.94	G238S	9.61	7.22	-2.74	negative
H153R	2.17	G238S	9.61	11.50	1.31	
**R164H**	**3.43**	**I173V**	**2.10**	**6.95**	**3.01**	**positive**
R164H	3.43	A224V	1.86	3.90	0.20	
**R164H**	**3.43**	**E240K**	**1.58**	**9.48**	**6.06**	**positive**
I173V	2.10	E240K	1.58	3.62	1.53	positive
**M182T**	**2.15**	**G238S**	**9.61**	**16.17**	**6.00**	**positive**
K215E	1.90	G238S	9.61	6.34	-3.58	negative
G238S	9.61	E240K	1.58	12.04	2.44	
G238S	9.61	T265M	N/A	10.84	N/A	

Mutated residues (columns 1 and 3) and their individual cefotaxime resistance levels (columns 2 and 4) are compared to resistance levels when they occur together in the same sequence (column 5). The level of cefotaxime resistance (an indicator of extended-spectrum antibiotic resistance) is shown in centimeters of linear growth on a 0.04 µg/ml cefotaxime gradient. The difference between the combined effect (column 5) and the sum of the individual effects (column 2 + column 4), which represents epistasis, is shown in column 6.

**Figure 4:**
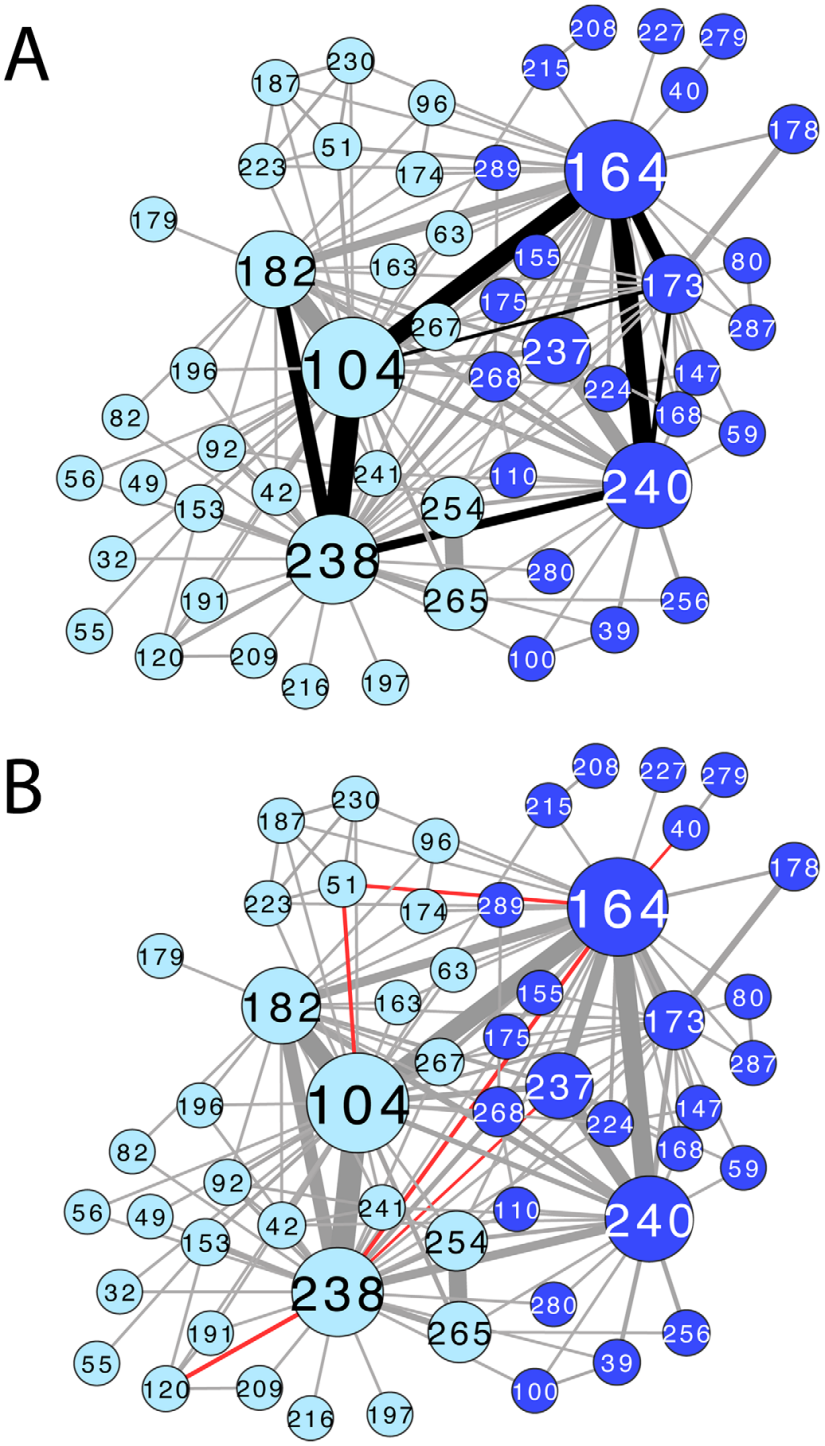
Pairwise epistatic interactions in the TEM extended-spectrum community either previously described in the literature or identified in our experiments [**19]**, [79]–[83****]. Network is represented as in Figure 2. The subcommunity containing the active-site residue 238 is light blue and the subcommunity containing the active-site residue 164 is dark blue. Node size is proportional to weighted degree centrality (Methods). Link thickness indicates how frequently two residues (nodes) are mutated in the same sequence, normalized by the number of sequences in which only one or the other position is mutated (Methods). (A) Black links indicate positive epistatic interactions. (B) Red links indicate negative epistatic interactions. Because the network is constructed from co-occurring mutated residue pairs, negative-epistatic pairs may be underrepresented in or absent from the network, e.g. 39 and 173. Image created with CytoScape [78].

*Significant epistatic effect  =  differences that exceed the margin of standard error (for the number of replicates (n), refer to Table S4). The sign of significant sign-epistatic interactions is shown in column 7. These interactions are mapped onto edges in our extended-spectrum community network (Figure 4A,B). Six interactions that were previously reported as positive are highlighted here in bold.

**Table 5:** Experimentally determined epistatic interactions between single mutations and mutation doublets in the extended-spectrum antibiotic resistance community network.

M1	M1 Growth [cm]	M2	M2 Growth [cm]	M1_M2 Growth [cm]	M1_M2 - (M1+M2) [cm]	Significant Epistastic Effect*
Q39R	2.09	E240K R164H	9.48	9.10	-0.88	
Q39R	2.09	E240K G238S	12.04	9.59	-2.95	negative
L40W	2.08	E104K R164H	8.42	5.06	-3.85	negative
L51P	1.93	M182T E104K	2.82	2.54	-0.62	
L51P	1.93	I173V R164H	6.95	1.79	-5.50	negative
L51P	1.93	G238S E104K	16.83	1.88	-15.29	negative
E104K	2.20	R164H L40W	2.13	5.06	2.32	positive
E104K	2.20	R164H A224V	3.90	9.31	4.80	positive
E104K	2.20	K215E G238S	6.34	11.26	4.31	positive
E104K	2.20	I173V R164H	6.95	16.49	8.93	positive
E104K	2.20	G238S T265M	10.84	19.40	7.95	positive
E104K	2.20	G238S H153R	11.50	17.65	5.54	positive
R120S	1.94	G238S H153R	11.50	14.36	2.51	
R120S	1.94	E240K G238S	12.04	12.92	0.53	
H153R	2.17	R120S G238S	7.22	14.36	6.56	positive
H153R	2.17	E104K R164H	8.42	11.50	2.50	positive
H153R	2.17	E104K I173V	10.84	2.65	-8.77	negative
H153R	2.17	M182T G238S	16.17	17.95	1.20	
H153R	2.17	E104K G238S	16.83	17.65	0.24	
R164H	3.43	Q39R E240K	2.16	9.10	5.10	positive
R164H	3.43	E104K H153R	2.73	11.50	6.93	positive
R164H	3.43	M182T E104K	2.82	16.86	12.20	positive
R164H	3.43	E104K I173V	10.84	16.49	3.81	positive
R164H	3.43	H153R G238S	11.50	6.00	-7.34	negative
I173V	2.10	R164H L51P	1.90	1.79	-0.62	
I173V	2.10	E104K H153R	2.73	2.65	-0.59	
I173V	2.10	E104K R164H	8.42	16.49	7.56	positive
I173V	2.10	E240K R164H	9.48	17.48	7.49	positive
M182T	2.15	E104K L51P	1.65	2.54	0.33	
M182T	2.15	E104K R164H	8.42	16.86	7.88	positive
M182T	2.15	G238S H153R	11.50	17.95	5.89	positive
K215R	1.90	E104K G238S	16.83	11.26	-5.88	negative
A224V	1.86	E104K R164H	8.42	9.31	0.62	
G238S	9.61	E104K L51P	1.65	1.88	-7.79	negative
G238S	9.61	Q39R E240K	2.16	9.59	-0.59	
G238S	9.61	K215R E104K	2.39	11.26	0.85	
E240K	1.58	Q39R R164H	3.70	9.10	5.41	positive
E240K	1.58	R164H I173V	6.95	17.48	10.54	positive
E240K	1.58	R120S G238S	7.22	12.92	5.71	positive
T265M	N/A	E104K G238S	16.83	19.40	N/A	

Mutated residues (columns 1) and residue pairs (column 3) and their corresponding cefotaxime resistance levels (columns 2 and 4, respectively) are compared to resistance levels when they occur together in the same sequence (column 5). The level of cefotaxime resistance (an indicator of extended-spectrum antibiotic resistance) is shown in centimeters of linear growth on a 0.04 µg/ml cefotaxime gradient. The difference between the combined effect (column 5) and the sum of the individual effects (column 2 + column 4), which represents epistasis, is shown in column 6.

*Significant epistatic effect  =  differences that exceed the margin of standard error (for the number of replicates (n), refer to Table S4). The sign of significant epistatic interactions is shown in column 7.

The observed disconnect between co-occurrence and the cefotaxime resistance phenotype of pairs of mutations included in our network suggests that the adaptive value of a given mutation or mutation pair is highly dependent on sequence context. Thus, an accurate assessment of the contribution of a given mutation to adaptation involves testing the effect of the mutation in the presence of different additional mutations, *i.e.* in a range of sequence contexts. Table 6 shows the impact of 14 of 16 mutations identified as of likely significance for extended-spectrum β-lactamase resistance based on shortest path betweenness centrality. Both the average effect (column 5) and the range of effects (in centimeters of continuous growth; column 6), obtained in a variety of sequence contexts, are shown. The number of sequence contexts tested (7 on average) is listed in column 4. The sequences tested and their measurements are listed in Table S4.

**Table 6:** Experimentally determined effects of individual mutated residue positions found in critical extended-spectrum antibiotic resistance network trajectories (Table 3).

Mutant position	Count within database	Mutation tested	Number of different sequence contexts tested	Average effect [cm]	Interval (min, max) [cm]
164	48	R164H	13	4.18	(-5.5, 14.04)
104	48	E104K	15	4.04	(-0.28, 9.54)
238	38	G238S	11	8.03	(0.23,14.63)
240	31	E240K	8	3.96	(0.07,10.53)
182	27	M182T	6	3.92	(0.62, 8.44)
265	20	T265M	2	1.90	(1.23, 2.57)
153	9	H153R	8	0.95	(-8.19, 7.14)
173	5	I173V	8	3.82	(-0.11, 8.64)
237	9	N/A	N/A	N/A	N/A
224	3	A224V	4	0.33	(-0.3, 0.89)
120	3	R120S	4	0.43	(-2.39, 2.86)
215	2	K215E*	4	-2.89	(-5.57, 0.19)
51	2	L51P	6	-3.69	(-14.95, 0.34)
268	2	N/A	N/A	N/A	N/A
40	1	L40W**	3	-1.39	(-3.36,0.49)
39	1	Q39R	6	-0.56	(-2.45, 0.58)

Critical triple mutant trajectories (Table 3) contain only 16 unique individual residue positions (column 1). The number of sequences in experimental and clinical isolates that have this residue position mutated is shown in column 2. For each residue position, we tested the most frequent amino acid substitution in these sequences, with two exceptions:

*: K215E has equal frequency to K215R and K215Q in the extended-spectrum phenotype sequence database;

**: L40W and L40V have equal frequencies (column 3). We tested the level of cefotaxime resistance of each mutation (centimeters of linear growth on a 0.04 µg/ml cefotaxime gradient) in a variety of sequence contexts. Each context consists of the relevant mutation plus different additional mutations, all of which are found in the critical triple mutant evolutionary trajectories. The number of sequence contexts tested is shown in column 4 and the different mutant combinations comprising each sequence context are shown in Table S4. Averaging the effect of each mutation across all its sequence contexts yields a measure of its global contribution to extended-spectrum antibiotic resistance (column 5). In general, the effects are highly dependent on sequence context, as shown by the wide range of outcomes (column 6).

Note that, in agreement with the epistatic analysis presented in Tables 4 and 5, most mutant positions exhibit a wide range of effects, and that these effects are frequently sign-epistatic (*i.e.* that, in addition to positive effects, include neutral and negative combined effects). The effect of the R164H mutation on cefotaxime resistance for example can go from −5.5 cm to +14.04 cm, that of H153R, from −8.19 cm to 7.14 cm. The average increase in cefotaxime resistance corresponds roughly to the count in our database, with frequent mutations (n> = 20) having a large impact on cefotaxime resistance (4.3±1.6 cm). H153R and I173V, two mutations with intermediate count (5<n<20), also have a clear impact on resistance, with maximal effects in the same order as those of frequent mutations. The average effect of infrequent mutations (n<5), by contrast, is negative (−1.3±1.6 cm), questioning the relevance of these mutations for extended-spectrum resistance. The large negative effects that some of these mutations — L51P (−14.95 cm), K215E (−5.57 cm); R120S (−2.39 cm) — have in specific contexts suggests that they are functionally important but that their effect is highly context-dependent. The two strongest negative epistatic effects we detected for infrequent mutations, those of L51P and K215E, are shown in Figure 3B.

## Discussion

Here we assembled a large database (n = 361) of mutants of the enzyme TEM-1 β-lactamase to study the genetic basis for adaptive evolution.

In the construction of this database we made the following two assumptions:

1) That most mutated positions would have undergone a degree of positive selection, which was supported by a PAML (codeml) analysis of clinical mutants (Table S3).

2) That experimental evolution is comparable to clinical evolution, given that both scenarios share a selective pressure (antibiotic resistance selection) and yield similar mutations [9].

We then used co-occurrence, *i.e.* presence of two mutations in the same sequence, as an indicator of potential functional interaction. Pairwise interactions were visualized using a network representation where each node is a mutant position, and each link represents occurrence of two mutated positions in the same sequence. The resulting undirected, weighted network has a few highly connected nodes and a majority of nodes exhibiting low connectivity (Figure S1). This connectivity property [53] is reminiscent of the link distribution in networks representations of other biological processes, such as cell signaling or differentiation, where it helps in buffering noise caused by random variation within the system. In the case of proteins, it may contribute to robustness to mutation.

We distinguished two levels of modular organization within our network of genetically defined interactions in TEM β-lactamase:

Large “communities”: These correspond to three distinct phenotypic categories: broad-spectrum, extended-spectrum and inhibitor resistance (Figure 1). The observed segregation of residue positions according to the selection driving their evolution is remarkable given that no phenotype class information was used to construct the network. This effect is consistent with previously described antagonistic pleiotropy between different resistance phenotypes [54]. Within the two adaptive communities (extended-spectrum and inhibitor resistance) we found that community annotation largely matched phenotypic data: Five mutant positions were correctly classified as inhibitor resistance mutations and 12 positions were accurately classified as extended-spectrum mutations (see legend to Figure 1). Interestingly, mutations that are known or suspected to contribute to both inhibitor and extended-spectrum antibiotic resistance (182, 268, 201) are at the interface between the two communities. Positions 100 and 147 are similarly located at this interface. These are positions with likely compensatory, thermodynamically stabilizing mutations [25], [26], [43], [51] that have been found in extended-spectrum evolution experiments too [19], [24], [36]. They may also belong to the dual resistance phenotype category, as experimental data on inhibitor resistance evolution is scarce. The only clearly misclassified mutant positions are: 175 (involved in extended-spectrum resistance [55] but classified as inhibitor resistance) and 130 (an inhibitor resistance mutation classified as broad-spectrum). In the case of the catalytic site residue 130, the misclassification was due to the fact that the S130G mutation confers resistance to inhibitors on its own and therefore rarely co-occurs with other mutations. Its assignment to the broad-spectrum community is based on a single co-occurrence event in our database.

“Subcommunities” within these communities: We hypothesize that subcommunities are likely to represent parallel strategies of adaptation within the community's phenotype class. This appears to be the case in the two adaptive phenotypic classes included in this study:

Inhibitor-resistance community: this network contains two subcommunities, corresponding to two distinct mechanisms disrupting inhibitor binding at the active site [56]. One involves positions 69 and 276, which are strongly connected in one subcommunity, and the other one involves 244, which is in a separate subcommunity (Figure 1).

Extended-spectrum resistance community: this network contains two large subcommunities (Figure 2). Central to each subcommunity is one position involved in substrate recognition, 164 and 238 respectively. R164H/S/C mutations are thought to lead to the collapse of the Ω-loop, creating greater active site accessibility (Figure S2A); G238S on the other hand, appears to increase affinity for the substrate and/or cause repositioning of the Ω-loop (Figure S2B). These two mutations were recently shown to represent alternative evolutionary solutions, leading to parallel, divergent mutation trajectories with different fitness optima [36]. In that study, divergent evolution appeared as a contingency effect of trajectories involving the negatively epistatic G238S or R164S mutations. Specifically, the first mutation significantly impacted the composition of subsequent evolutionary trajectories [36]. In our network analysis, divergent evolution is represented by the two subcommunities defined by residues 164 and 238. Most nodes have strong connections (high-weight links) to one of these subcommunities and much weaker connections (very low-weight or absent links) to the other subcommunity. For example, position 237 is strongly linked to 164, but is weakly connected to nodes from the 238 subcommunity. This non-uniform node connectivity agrees with a recent laboratory evolution study [36], which reported that E104K is preferentially selected in G238S trajectories, while E240K is more frequently found in R164S trajectories. Therefore, our network can be used to make inferences on evolutionary contingency effects, at least for the two main fitness peaks present in extended-spectrum evolution. The observation that other residue positions are frequently linked with both 164 and 238 in our network, even if we typically find a preference for one or the other, indicates that the evolutionary divergence associated with the two fitness peaks is only partial.

In sum, we find that both distinctive selective pressures and peaks within the enzyme's fitness landscape leave recognizable footprints on the network's connectivity. Furthermore, the amino acid positions within network modules are not necessarily physically close in the protein's tertiary structure, as interactions are defined genetically (functionally) rather than physically. To illustrate this point, Figure S3 maps nodes (mutant positions) belonging to the three major communities in the TEM coevolution network (Figure S3A) and the two main extended-spectrum resistance subcommunities (Figure S3B) onto the tertiary structure of the TEM enzyme (PDB ID: 1ero). It is apparent that neither community is physically localized to a defined area of the protein.

Link weights in our network are proportional to the number of sequence co-occurrence events for the corresponding mutated residue pairs. We implicitly incorporated epistatic information into this metric by using a normalization factor: We compared the number of mutated position pair occurrences with the mutation count at each of the corresponding individual residues (Methods, Equation 1). In addition, we calculated the difference between the raw co-occurrence weights and weights normalized in this way (Table S5): A positive difference indicates a trend toward positive epistasis between the two residue positions involved, whereas a negative difference is indicative of possible negative epistasis. Positive epistasis trends predicted in this way appear to be in agreement with experimentally proven epistatic interactions: 10 interactions were described as such in the literature, and only 2 cases, both involving position 237 (Table S5), have been reported as negative. The negative difference also correctly predicts the negative-epistatic interactions within residue pairs 173–182 and 164–238. Negative pairwise interactions are, however, underrepresented in the input to our network because mutations at these residue positions are infrequently selected together.

By connecting individual nodes (representing mutated residue positions), paths through our network define potential evolutionary trajectories. Network metrics allowed us to extend the trajectories beyond the pairs of co-occurring nodes used to build the network. We focused on combinations of three mutations, which are the most experimentally tractable ones. Our basic hypothesis was that genetic adaptation necessitates a specific combination of functional milestones, where each amino acid mutation represents a potential milestone. According to this hypothesis, combinations of mutations that facilitate information flow through the network should contribute prominently to genetic adaptation. We used shortest path betweenness centrality (a metric that can be interpreted as measuring a path's importance for information flow within the network) to identify trajectories of potential special significance for extended-spectrum β-lactamase resistance (Table 2). The following points support the special significance of triple mutant trajectories with nonzero betweenness centrality:

- They occur frequently in natural or experimental extended-spectrum β-lactamase evolution experiments (Table 2, column 4).
- The higher the betweenness centrality, the more likely they are to have been previously seen (Table 2).
- Presence of these mutations in reported (previously seen) sequences is associated with increased cefotaxime resistance, an indicator of extended-spectrum activity (Table 3).

All predicted triple mutant combinations that were experimentally tested and that significantly improved resistance over constituent mutant pairs (a total of 8) have been previously described. Of these, only two (M182T G238S H153R and E104K R164H A224V) were absent from our original database and have been reported only recently [36]. Their impact on cefotaxime resistance is shown in Figure 3A.

By construction, the network only contains information about mutation pair occurrence counts (regardless of whether the pairs are components of more complex mutant sequences). Therefore, all mutation triplets with increased resistance constitute predictive successes, regardless of whether or not sequences containing these mutations were part of the original database. We used the strong association between previous observation of a TEM mutant and its increased resistance to estimate our success rate at 23 out of 48. As a control, we ran a computational simulation to find the success rate we would have obtained by random sampling from positions involved in extended-spectrum resistance weighted by residue mutation frequency in our database. The average result of 10,000 random samplings was 12.8±3.08 out of 48, proving that our method is able to extrapolate triple mutant trajectories from pairs of coevolving mutations more accurately than simply combining mutations of high frequency.

At this time, the predictive value of our method can only be rigorously supported with respect to known TEM mutant combinations. *A priori*, there is no reason to believe that our method cannot find adaptive combinations of mutations that have never been seen before. However, the success rate for new sequence space should vary substantially between genes, depending on how extensively they have already been sampled by natural and/or experimental evolution.

Our method for identification of paths of special significance for adaptation has limitations, because it assumes that each mutant position has a discrete effect on adaptation and that this effect is sufficiently unique that adaptation requires a composite solution. Therefore, global suppressors (such as mutations at position 182) or mutations with a large impact on their own (S130G, associated with inhibitor resistance, and G238S conferring extended-spectrum resistance) will not be adequately accounted for by our “information flow” metric.

Another example of this method's limitations is illustrated by the absence of the high fitness extended-spectrum triple mutant 104-238-182 in our list of nonzero betweenness centrality triplets (Table 2). Amino acid substitutions at 104-238-182 were the most frequent combination obtained from TEM-1 libraries subjected to cefotaxime selection in a recent study [36]. The presence of a global suppressor (182) and of a mutation with a large impact on its own (G238S) likely explains why this triple mutant combination is not among the nonzero betweenness paths in Table 2. However, parallel, divergent evolutionary trajectories identified by this study are enriched for triple mutant trajectories with high betweenness centrality (detailed in Table S6 and Text S1 Results). Overall, triple mutant trajectories with nonzero betweenness centrality are frequently contained within mutational trajectories parallel to E104K M182T G238S. Thus, our method is able to identify paths of special significance for genetic adaptation, although with decreased sensitivity to mutations with a large impact on their own and to global suppressors.

Next, we investigated whether links connecting co-occurring pairs in our network represent positive functional interactions. We tested the individual vs. combined effects of the mutations in the mutant triplets from Table 3. The results are listed in Table 4: A difference between adding the individual fitness effects of two mutations (M1+M2) and the combined fitness effect of the double mutant (M1_M2) is indicative of epistasis. This table includes six interactions previously reported as positive epistatic (highlighted in bold). In agreement with previous reports, our experiments show significant positive epistasis in all cases but E104K M182T, which in our hands is simply additive. We also found two new examples of positive epistasis involving I173V (E104K I173V and II73V E240K), and we identified five new examples of negative epistasis. The high count of negative interactions in our tested pairs is surprising given that each connected pair of nodes represents pairs of mutations that co-occur in at least one sequence. We assume that the reported sequences containing these negatively epistatic mutations must have additional mutations producing an overall positive outcome. Similarly, we found a number of significant negatively epistatic interactions in the triple mutants tested (8 out of 27; Table 5). Thus, even in a network model representation intrinsically biased against negative epistasis, we frequently identify negative epistatic interactions among linked mutation pairs. This observation highlights the pervasiveness of negative epistasis in TEM extended-spectrum resistance evolution and shows that links within our network are more indicative of potential functional interactions than of positive epistasic interactions.

Figure 4 maps experimentally determined pairwise epistatic interactions (either from previous reports in the literature or from this study) onto the TEM extended-spectrum coevolution network: Figure 4A shows the positively epistatic interactions, and Figure 4B shows negatively epistatic interactions. Some known negative epistatic interactions may, however, be absent from our network representation, as they would rarely co-occur in the same sequence.

The 48 triple mutant paths we identified as of special significance (Table 2) consist of different combinations of only 16 residue positions (listed in Table 6, column 1). These include 10 positions with a demonstrated effect on extended-spectrum β-lactamase resistance, out of 12 known to date [9]. The two false negatives are positions 175 and 179, each of which arises independently only once (Text S1 Methods) in our extended-spectrum sequence database. 175 is one of a number of positions in the Ω-loop (involved in active-site formation) that are known to play a role in extended-spectrum resistance [9], [55]. 179 was previously reported in a clinical isolate [20], [57] and in several experimental isolates [58] but appears to have a narrower substrate specificity than other mutations present in the extended-spectrum network community [9]. Our analysis suggests that the remaining 6 mutant positions present in the nonzero betweenness triple mutant paths (40, 120, 153, 215, 224, and 265) should be considered as potentially important for adaptation.

We evaluated the relevance of 14 out of the 16 positions identified by our betweenness centrality analysis by experimentally determining their cefotaxime resistance phenotype. In order to factor in the prevalent role of epistasis in extended-spectrum TEM evolution, we determined the impact of a given mutation on cefotaxime resistance as the average phenotype in a variety of sequence contexts (in the presence of a variety of additional mutations). The results are summarized in Table 6, with the number of sequence contexts tested listed in column 4, the average effect (in cm) shown in column 5, and the range of effects listed in column 6. Our results show a relationship between average phenotypic effect and representation in our database, with frequent mutations (n>4) having a clear average positive effect (> = 1 cm). Our phenotypic analysis also confirms the relevance of mutations at three positions whose phenotypic impact on extended-spectrum resistance had, to our knowledge, not been previously demonstrated: 265 (average 1.9 cm, up to 2.6), 153 (average 1.0 cm, up to 7.1), and 120 (average 0.4 cm, up to 2.9). The effect of 153 is strikingly sequence-context dependent, with values ranging from −8.19 to +7.14 cm, which may explain why the role of this mutation has been hard to experimentally demonstrate. R164H and L51P, two mutations with a known effect on resistance phenotype, had large negative impacts in some sequence contexts: −5.5 and −14.95 cm, respectively. These observations imply that a strong negative epistatic effects may be as indicative of functional interactions as a positive epistatic effects. Therefore, the large negative effects K215E (−5.57 cm) and L40W (−3.4 cm) suggest an important role for these residue positions that is only revealed in specific sequence contexts, although this remains to be experimentally confirmed.

In sum, positions present in triple mutant paths with nonzero betweenness centrality identified all but two of the positions with known phenotypic effect on extended-spectrum resistance. We experimentally demonstrated the impact of the additional mutations identified by our analysis, either directly by showing increased cefotaxime resistance (120, 153, 265) or indirectly, by showing large negative effects on resistance (215 and 40). These results suggest that our method is able to accurately identify positions that play an important role in genetic adaptation. It is able to do so because it evaluates mutations in the context of their genetically defined functional interactions.

Many current state-of-the-art bioinformatics methods for predicting mutation effects consider only evolutionary history and/or biophysical properties of single residue positions [59], [60]. Previous methods that consider interactions among residue positions include evolutionary trace, statistical-coupling and residue coevolution networks [61]–[64]. Evolutionary trace (ET) [61] uses a phylogenetic tree to group protein sequences and rank the functional importance of amino-acid residues by correlating their evolution with divergence in the tree. Residues traced in this way are mapped onto a protein structure, and sites of clustering can be used to infer functionally important sites. Statistical coupling analysis [62] relies on partitioning and perturbation of large and diverse multiple sequence alignments of homologous proteins to study higher-order interaction patterns. More similar to our approach are two previous studies of protein residue coevolution networks, based on large, diverse protein families. They found that node connectivity and centrality had utility in predicting functionally important residues [63] and that functionally important residues tend to coevolve with other sites more than other residues [64].

In contrast to these methods, our approach uses network analysis to infer higher-order evolutionary interactions between groups of coevolving residues that may not be co-localized in a protein structure. Our focus is not on finding functionally important residues. Rather, we identify communities of residue positions associated with different antibiotic resistance phenotypes and subcommunities representing distinct strategies to acquire a given resistance phenotype. We are also able to extrapolate adaptive evolutionary trajectories – combinations of triple mutants that increase cefotaxime resistance – based only on the initial knowledge of the co-occurrence of mutated residues in resistant mutant sequences. Our method can be applied to protein subfamilies in which there is low sequence diversity, and it does not require a reliable phylogeny or tertiary protein structure on which to base inference.

While we use TEM β-lactamase as a model system in this paper, we believe that our network analysis is generalizable to other genes evolving under defined selective pressures. This model presents a desirable alternative to phylogeny in many situations, *e.g.* genes in clinical isolates for microorganisms with frequent horizontal transfer or/and high geographic mobility, and microorganisms with high genetic variation, where positively selected mutations are in the minority, such as RNA viruses. If however, reliable phylogenetic data is available, phylogeny could be incorporated into our analysis to help estimate how many times pairs of mutated residues arise independently and to reveal the ordering of mutation events. The construction of a directed rather than undirected network model could enable us to better project the order of mutations in future predicted trajectories.

## Materials and Methods

### Data collection

We compiled a set of 363 TEM mutant protein sequences from existing databases and literature: the Lahey Clinic β-lactamase database [10], the Lactamase Engineering Database (LacED) [11], and from published directed evolution experiments that select for TEM mutants exhibiting various resistant phenotypes [12]–[26].

### Sequence alignment

Using TEM-1 as the reference sequence [10] and the Ambler TEM amino acid residue numbering scheme [65], we constructed a multiple sequence alignment of naturally occurring and laboratory-evolved TEM mutants (Tables S1 and S2). To estimate the number of times that mutations at two residue positions have coevolved, we counted independently selected mutation pairs (Text S1 Methods). FASTA-formatted TEM mutant sequences are provided as Datasets S1-S8.

### TEM coevolution network construction

We constructed an undirected, weighted network in which two nodes (two mutated amino acid residue positions) are linked if mutations at both residues exist in at least one TEM sequence in the alignment. The weight *w* of each link is proportional to the number of sequences in which both positions are mutated, normalized by the number of sequences in which only one or the other position is mutated:
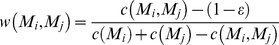
(1)where *c(M_i_)* and *c(M_j_)* are the number of times a the ith and jth column (residue position), respectively, are mutated in the alignment. *c(M_i_,M_j_)* is the number of times both columns are mutated together, and *w(M_i_,M_j_)* is the network weight of the link between nodes *i* and *j* (or residue positions *i* and *j*).

We included a correction term to ensure that mutated pairs, which occur in a single sequence together and never by themselves, are not overweighted. Without this term, these pairs would always have (the maximum) link weight 1.0. *ε* is the inverse of the number of aligned sequences used to construct the network (a heuristic choice that works well in practice).

### Annotation of TEM sequences by their phenotype class

We were able to associate 380 out of 405 TEM naturally occurring or TEM laboratory-evolved mutant sequences in our database with a single major β-lactamase phenotype class (113 broad-spectrum 2b sequences, 201 extended-spectrum 2be sequences, 49 inhibitor-resistant, 2br, sequences). There were also 17 sequences with a combined extended-spectrum antibiotics and inhibitor resistant phenotype class, 2ber, that were not used in our network. The phenotype class of naturally occurring TEMs is determined experimentally, and TEM sequence-to-phenotype-class associations can be found in the Lahey Clinic β-lactamase online database [10]. We assumed that the resistance selection criterion used in the directed evolution experiments [12]–[26] determined the phenotype class of the TEM sequences coming from such experiments.

### 2be phenotype class network construction

To explore the subcommunity structure of the 2be phenotype class, we constructed an undirected, weighted coevolution network (as above), using only 201 (naturally occurring and laboratory-evolved) extended-spectrum sequences.

We observed a few differences between the wiring of the extended-spectrum phenotype network and its corresponding community in the TEM coevolution network. In the first case, all residue positions that can be associated with extended-spectrum resistance were included in the sequences used to build the network. In the second case, some mutated residue positions (as opposed to mutant sequences) can be associated with more than one phenotype class (pleiotropy). However, by construction, the community-finding algorithm associates the corresponding nodes with only one community. These differences were minor and did not have an impact on the conclusions of our analysis.

### Network analysis

#### Network modularity

To identify highly connected subnetworks (communities) of mutated residue positions, we used the Community-Structure-Partition algorithm [31], implemented in the Graph Utilities Package in Mathematica 7.0 [66]. Communities with five or fewer nodes were merged onto one of the larger communities. The choice of a larger community onto which to merge the smaller community was determined by calculating the overall network modularity function [31] after a suggested merge. The merge that resulted in the highest network modularity was the one that was chosen.

#### Central nodes in the network

We used three standard graph-theoretical node centrality metrics to identify important residue positions in our undirected, weighted network: degree centrality, closeness centrality and betweenness centrality. To calculate the closeness and betweenness centrality metrics, we transformed link weights into link costs by taking the inverse of each pair association weight. A detailed description of all node centrality metrics used in our study can be found in Text S1 Methods.

#### Length of shortest paths

A path is a set of adjacent links in a network, which connects a pair of nodes *v* and *w*. The length of the shortest path between two nodes *dG(v, w)* in a weighted network is the minimum sum of link costs along an optimal path between nodes *v* and *w*. We compute link costs as the inverse of link weights.

#### Path betweenness centrality

We adapted the equation for weighted node betweenness centrality (Equation S3) to multiple-node path betweenness centrality
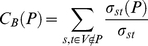
(2)where *P* is a path comprised of distinct adjacent nodes in the network, and *σ_st_(P)*, is the number of distinct shortest paths connecting the network nodes *s* and *t* and containing *P* as a subpath. This metric counts the number of times a subpath occurs as a component of all shortest paths between all pairs of nodes in a network.

### Codon-model analysis of selection

We performed a PAML (codeml) analysis [67] for the naturally occurring TEM β-lactamase sequences. We used PHYLIP [68] to build a phylogenetic tree (gamma distribution, four classes, α parameter: 0.348). We used a log-likelihood test to compare the fit of codeml models 2 (three-classes of unselected/selected codon positions) and 1 (two-classes of unselected/selected codon positions) to the data, and found that model 2 was a better fit (χ2 test, p-value <<0.01). Using model 2′s three site classes, we found that out of 35 mutated residue positions in the network of naturally occurring TEM sequences, 11 are identified as strongly positively selected (ω > = 8.4) and 22 are positively selected (relaxed to ω> = 0.8) (Table S3).

### Experimental tests of TEM mutants

#### Selection of mutants to be tested

48 out of a possible 214 three-node shortest paths in the 2be community network had nonzero betweenness centrality, and we focused our experiments on the corresponding 48 triple mutants. Because all triplets represent a mutational trajectory and are therefore ordered, we compared the activity of each triplet to each possible trajectory (*i.e.* doublet) that led to it.

#### Site-directed mutagenesis of TEM β-lactamase

Our target TEM-1 β-lactamase sequence is in pGPS-ori, a pGPS3-derivative with a β-lactamase gene moved close to the origin of replication and with kanamycin as selectable marker [69]. We used previously described β-lactamase mutants generated under aztreonam selection [18]. Additional mutants were generated using the “megaprimer” protocol [70]. Briefly, we amplified the β-lactamase gene with a forward or reverse primer bearing the desired point mutation and an additional 5′ or 3′ flanking primer (all primers used are listed in Table S7). The PCR product was purified by gel electrophoresis and served as a “megaprimer” for whole plasmid amplification in the next PCR reaction, using pGPSori as the template. Standard PCR conditions apply, except elongation time is extended to 2 minutes/kb. When the reaction is complete, the PCR product is treated with the restriction enzyme DpnI, which digests only methylated (template) DNA. 8 ul of the megaprimer product are transformed into chemically competent TOP10 cells (Invitrogen) and selected on kanamycin (30 ug/ml). Incorporation of the intended point mutations was verified by sequencing of purified plasmids. All DNA isolation procedures were performed with Machery Nagel's Nucleospin Plasmid miniprep kit. Sequencing was carried out by Sequetech (Mountain View, CA).

#### Cefotaxime protection

We found that JS200 (a B strain of E. coli) was more sensitive to cefotaxime than BL21, which is a standard K strain (not shown). Therefore we used JS200 (SC-18 recA718 polA12ts uvrA155 trpE65 lon-11 sulA1) cells complemented with pHSG-Pol I plasmid as hosts [71]. To determine extended-spectrum β-lactamase protection, we used cefotaxime as antibiotic of choice, following the example of previous experimental studies of extended-spectrum β-lactamase evolution [9], [24]. Given the large number of clones involved in our study, we established a gradient plate assay to determine cefotaxime resistance levels in an efficient manner. This assay is conceptually similar to inhibition zone assays, but produces growth rather than absence of growth as output. This method is described in detail in [72]. Briefly, 25 ml LB agar containing a given concentration of cefotaxime is poured on a tilted plate and allowed to solidify. Then the plate is placed flat on a surface and drug-free agar is poured on top, creating a gradient of drug concentrations along the length of the plate. Individual β-lactamase mutants were transformed and individual colonies were grown to late exponential phase. We tested 3 to 4 independent clones for each mutant, and frequently more. All the measurements are listed in Table S4, with the number of measurements shown in column AI. Cultures of transformant cells were stamped on LB agar plates containing cefotaxime gradients and grown overnight at 37°C. The next day the plates were imaged and length of continuous growth along the drug gradient (in centimeters) was measured; a detailed description of this technique can be found in [72]. Given the wide range of resistance phenotypes present in our mutant panel, we tested each clone in gradients containing different cefotaxime concentrations to find one concentration providing adequate resolution. Wild-type TEM and a TEM-deleted plasmid (delta) provided good resolution at gradients containing a maximal concentration of 0.04 ug/ml (Table S4). Additional concentrations used were: 0.08, 0.12, 0.3, 0.6, 2 and 4 ug/ml (Table S4). Our negative controls were pGPSori and a plasmid encoding no β-lactamase (delta).

#### Quantification of cefotaxime protection

Our gels produced reproducible measurements, with an average standard error of 19% for all the 58 clones tested at the optimized concentration of cefotaxime (Table S4). In addition, each gel had one clone of known level of resistance as a control. We also had to find a way compare results from gradients containing different drug concentrations because, as mentioned above, the concentration of cefotaxime necessary to produce the intermediate level of growth in our gradient required for optimal resolution varied substantially depending on the level of resistance of each individual clone. To that end, we measured the growth distance for a number individual reference clones at two contiguous concentrations and averaged the difference; this average difference was used to extrapolate measurements to the concentration used to run the wild-type and clones with low levels of resistance (0.04 ug/ml); the number of clones tested for each conversion step (n), the difference between contiguous concentrations, and the aggregate average difference (conversion factor) are listed in Table S8. Note that the introduction of this conversion factor should not affect comparisons between clones showing similar levels of resistance, since these would have been run in gels with the same drug concentration; its purpose is to allow comparisons between clones showing substantial differences in level of resistance and should therefore not alter the results qualitatively.

## Supporting Information

Figure S1 **The weighted degree distribution of the TEM coevolution network (** **Figure 1** **).** The distribution of nodes by aggregate weight of links per node (weighted degree centrality, Equation S1) is shown. Many nodes (residue positions) with high weighted degree are functionally important (Table 1). The distribution reveals that the network contains very few highly connected nodes, with a majority of the nodes exhibiting low connectivity. This topology is similar to that of scale-free networks [53], and is reminiscent of the connectivity distribution of other biological processes such as signaling or cellular differentiation.
(TIF)

Figure S2 **Structural impact of extended-spectrum antibiotic resistance mutations.** (A) Mutations at residue 164. An arginine to serine (or arginine to histidine) substitution at position 164 (blue spheres) has been hypothesized to collapse the critical Ω-loop (green) in the active site, thus opening the active site to β-lactams with larger side chains [9], [74], [77] (PDB ID [84]). The ligand (shown in stick representation) is an N-Formimidoyl-Thienamycine pseudo-substrate from PDB ID 1jvj [85]. (B) Mutations at residue 238. A glycine to serine (or glycine to alanine) substitution at position 238 has been hypothesized to expand the active site by either repositioning the B3 β-strand (positions 235-240) [73] (yellow) or by tilting the Ω-loop (green) (positions 161-179) [75] that connects the two sub-domains of the protein. Mutations at both positions are associated with increased resistance to third generation cephalosporins [38].
(TIF)

Figure S3 **Locations of amino acid residues in the TEM coevolution network and the TEM extended-spectrum community network, mapped onto the TEM tertiary structure (PDB 1ero).** (A) Residues in the TEM coevolution network and their three major communities (Figure 1). Residues are colored by community membership: gray (broad-spectrum resistance), blue (extended-spectrum resistance) and orange (inhibitor resistance). The communities do not map to distinct regions of the tertiary structure. Image created with UCSF Chimera [86]. (B) Residues in the TEM extended-spectrum community network and their two major subcommunities (Figure 2). Residues are colored by subcommunity membership: light blue (subcommunity containing the active-site residue 238) and dark blue (subcommunity containing the active site residue 164). The subcommunities do not map to defined regions of the tertiary structure. Image created with UCSF Chimera [86].
(TIF)

Table S1 **Lists of all** ***naturally occurring*** **TEM-1 mutants in our database.** Each mutant is listed in column 1 and amino acid substitution mutations are indicated by residue position according to the Ambler system [65]. The mutants are listed in three worksheets by Bush-Jacoby phenotype class [27]: broad-spectrum antibiotic resistance (2b), extended-spectrum antibiotic resistance (2be), and inhibitor resistance (2br). FASTA sequences for all TEM-1 mutants from clinical isolates are included in Datasets S5-S8.
(XLSX)

Table S2 **Lists of all** ***laboratory-evolved*** **TEM-1 mutants in our database.** Each mutant is listed in column 1, and amino acid substitution mutations are indicated by residue position according to the Ambler system [65]. The mutants are listed in three worksheets by Bush-Jacoby phenotype class [27]: broad-spectrum antibiotic resistance (2b), extended-spectrum antibiotic resistance (2be), and inhibitor resistance (2br). References for each directed evolution experiment are provided in Dataset S4. FASTA sequences for all TEM-1 mutants from laboratory-evolved isolates are included in Datasets S1-S3.
(XLSX)

Table S3 **Codon-based analysis of positive selection.** We performed a PAML (codeml) [67] analysis for the naturally occurring sequences. Residue position number according to the Ambler system [65] (column 1); wild-type amino acid residue in TEM-1 (column 2); ω value (ratio of non-synonymous to synonymous nucleotide substitutions at a codon position) (column 3). "Site class" identified by codeml. Class 3  =  strong positive selection, Class 2  =  "relaxed" positive selection, Class 1  =  no selection or negative selection (column 4). Node degree centrality  =  weighted degree centrality computed in a network constructed with only sequences from clinical isolates (column 5).
(XLSX)

Table S4 **Cefotaxime gradient measurements.** All mutants and controls tested experimentally for cefotaxime resistance are listed in column A. The concentration empirically found to produce adequate resolution (i.e. intermediate level of growth in the gradient) is listed in column B. Columns C through AF list measurements (in centimeters) of continuous growth at the optimized concentration. The limit of continuous growth at the optimized cefotaxime concentration is listed (in centimeters). The average of all the measurements for a given clone is shown in column AG, with the corresponding standard deviation, standard error and % standard error in columns AH, AJ, and AK respectively. The total number of measurements (n) for each clone is provided in column AI.
(XLSX)

Table S5 **Network link weight normalization is predictive of epistasis.** Links between nodes were weighted according to the frequency in which the relevant pair (i.e. the mutant positions joined by the link) occurred in our database. This metric was further modified using a normalization factor comparing frequency of co-occurrence to that of the corresponding individual mutations. The two positions are listed as M1 and M2 in columns 1 and 2. The difference between observed co-ocurrence in the same sequence and the predicted frequency based on the frequency of each individual mutation constituting the pair is shown in column 3 (“predicted interaction based on link-weight normalization"). A positive difference indicates a trend toward positive epistasis between the two residue positions involved, whereas a negative difference of is indicative of possible negative epistasis. Column 4 indicates whether we found the interaction to be significantly epistatic in our cefotaxime resistance assays (Table 4), and column 5 lists the experimentally determined trend. Column 6 lists demonstrated epistatic interactions reported in the literature, and column 7 lists the corresponding references (the full reference is found at the bottom of the worksheet).
(XLSX)

Table S6 **Presence of triple mutants predicted to be of special significance for adaptation within cefotaxime-driven evolutionary trajectories.** Cefotaxime-driven evolutionary trajectories reported by Salverda *et al.* [36] are listed in column 2. This work investigated the role of contingency of the first mutation to be fixed under a given selective pressure on subsequent evolution. The clone number for each independent isolate, reported in [36] (figures 2A, 3A and 5A), is listed in column 1. Each directed evolution experiment is listed on a separate tab of this worksheet, with the sequence used to generate the original libraries for each of these experiments listed in column 3: *TEM-1 evolution*: 7 trajectories (out of 12) contained the triple combination of E104K M182T G238S mutations, and the 5 remaining trajectories represent parallel evolutionary trajectories; *R164S evolution; A237T evolution; R164S/G238S evolution; A237T/G238S evolution*. The triple mutant combinations present in the reported sequences and corresponding to evolutionary trajectories of special significance listed in Table 2 are highlighted in bold in column 2 and listed in column 4. Betweenness centrality values, indicative of the amount information flow (Methods), for trajectories of special significance are listed in column 5 and the average betweenness centrality values per clone are shown in column 6.
(XLSX)

Table S7 **Primers used for site-directed mutagenesis of TEM β–lactamase.** The primers used for site-directed mutagenesis by the megaprimer protocol [70] are listed, grouped by orientation. For each primer, the name, sequence, and amino acid substitution are shown.
(XLSX)

Table S8 **Calculation of conversion factor used to extrapolate growth to the gradient concentration used for wild-type.** A conversion factor was used to compare results from gradients containing different drug concentrations. To that end, we measured the growth distance for a number individual reference clones at two contiguous concentrations and averaged the difference. This average difference was used to extrapolate measurements to the concentration used to run the wild-type and clones with low levels of resistance (0.04 ug/ml). The number of clones tested for each conversion step (n) is listed in column 2, the difference between contiguous concentrations in column 3, and the aggregate average difference (conversion factor), in column 4.
(XLSX)

Table S9 **Calculation of average effect of mutations at individual positions tested in a variety of sequence contexts.** 14 out of 16 positions identified in high betweenness centrality trajectories were tested experimentally in a variety of sequence contexts. For each position, the most frequent mutation found in our database was used (see Table 6). The position being tested (M1) is listed in column 1, and its count in our database in column 2. Additional mutations (M2) are listed in column 3. The growth, in centimeters (extrapolated to a cefotaxime gradient of 0.04 ug/ml, see ) is listed in columns 3 (M1), 6 (M2) and 8 (M1+M2). The difference between M1+M2 and M2 measurements, representing the effect of M1 on resistance, is listed in column 11. We conservatively estimated the combined standard error to be the sum of the standard errors (p<0.05) of M2 and M1+M2 (column 10). The effect of M1 was considered to be significant (p<0.05) when the value for the combined margin of error was smaller than the p-value of the effect (column 12). Columns 13-15 list the average for each M1 position, the maximal positive effect, and the maximal negative effect, respectively. These values are listed also in Table 6 of the main text, columns 5 and 6.
(XLSX)

Text S1 **Supporting Methods and Results sections.** The *Methods* section outlines important processing steps for TEM mutations before their inclusion in the network. Key node network centrality metrics are also presented. The *Results* section highlights the observation that the evolutionary trajectories we predicted by our betweenness centrality ranking are enriched in paths that are parallel to the 104-238-182 evolutionary trajectory.
(DOC)

Dataset S1 **FASTA formatted protein sequences used in the construction of the TEM coevolution network.** Sequences are collected from published laboratory evolution experiments selecting for resistance to extended-spectrum β-lactam antibiotics.
(FA) Click here for additional data file.

Dataset S2 **FASTA formatted protein sequences used in the construction of the TEM coevolution network.** Sequences are collected from published laboratory evolution experiments selecting for resistance to β-lactamase inhibitors.
(FA) Click here for additional data file.

Dataset S3 **FASTA formatted protein sequences used in the construction of the TEM coevolution network.** Sequences are collected from published laboratory evolution experiments selecting for resistance to broad-spectrum β-lactam antibiotics.
(FA) Click here for additional data file.

Dataset S4 **References for the laboratory-evolved TEM sequences in Datasets S1 through S3.**
(XLSX)

Dataset S5 **FASTA formatted protein sequences used in the construction of the TEM coevolution network.** Sequences are collected from clinical isolates that have been demonstrated to confer resistance to extended-spectrum β-lactam antibiotics.
(FA) Click here for additional data file.

Dataset S6 **FASTA formatted protein sequences used in the construction of the TEM coevolution network.** Sequences are collected from clinical isolates that have been demonstrated to confer resistance to β-lactamase inhibitors.
(FA) Click here for additional data file.

Dataset S7 **FASTA formatted protein sequences used in the construction of the TEM coevolution network.** Sequences are collected from clinical isolates that have been demonstrated to confer resistance to broad-spectrum β-lactam antibiotics.
(FA) Click here for additional data file.

Dataset S8 **FASTA formatted coding DNA sequences used in the TEM phylogenetic analysis.** Sequences are collected from clinical isolates that have been demonstrated to confer resistance to extended- or broad-spectrum β-lactam antibiotics, or to β-lactamase inhibitors.
(FA) Click here for additional data file.
