# Supplementary figures and images for: Network Models of TEM β-Lactamase Mutations Coevolving under Antibiotic Selection Show Modular Structure and Anticipate Evolutionary Trajectories
Source: PLoS Comput Biol. 2011 Sep 22;7(9):e1002184. doi: 10.1371/journal.pcbi.1002184 (PMC3178621; doi:10.1371/journal.pcbi.1002184)

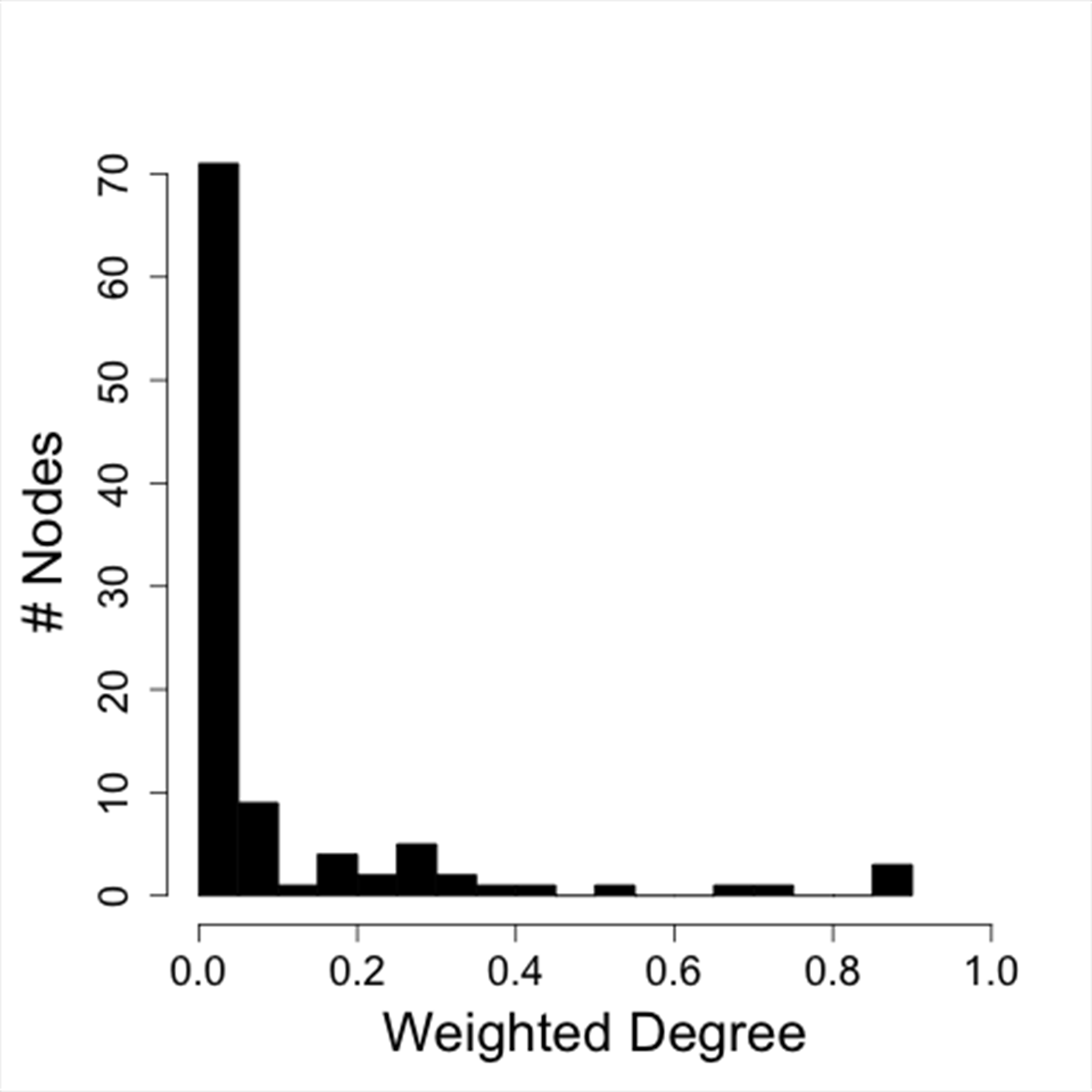

Supplement: Figure S1 — The weighted degree distribution of the TEM coevolution network ( Figure 1 ). The distribution of nodes by aggregate weight of links per node (weighted degree centrality, Equation S1) is shown. Many nodes (residue positions) with high weighted degree are functionally important (Table 1). The distribution reveals that the network contains very few highly connected nodes, with a majority of the nodes exhibiting low connectivity. This topology is similar to that of scale-free networks [53], and is reminiscent of the connectivity distribution of other biological processes such as signaling or cellular differentiation. (TIF) [file pcbi.1002184.s001.tif]

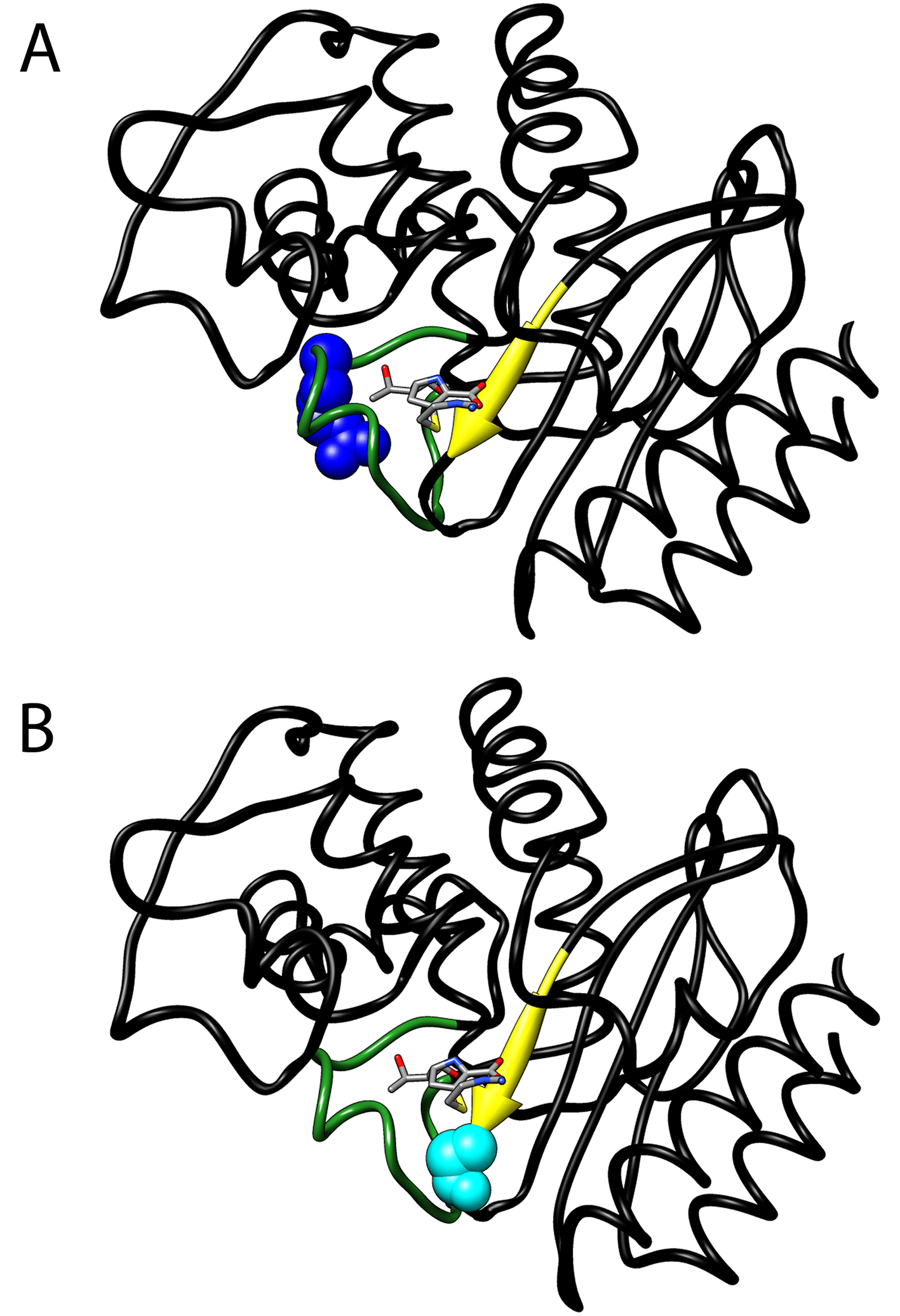

Supplement: Figure S2 — Structural impact of extended-spectrum antibiotic resistance mutations. (A) Mutations at residue 164. An arginine to serine (or arginine to histidine) substitution at position 164 (blue spheres) has been hypothesized to collapse the critical Ω-loop (green) in the active site, thus opening the active site to β-lactams with larger side chains [9], [74], [77] (PDB ID [84]). The ligand (shown in stick representation) is an N-Formimidoyl-Thienamycine pseudo-substrate from PDB ID 1jvj [85]. (B) Mutations at residue 238. A glycine to serine (or glycine to alanine) substitution at position 238 has been hypothesized to expand the active site by either repositioning the B3 β-strand (positions 235-240) [73] (yellow) or by tilting the Ω-loop (green) (positions 161-179) [75] that connects the two sub-domains of the protein. Mutations at both positions are associated with increased resistance to third generation cephalosporins [38]. (TIF) [file pcbi.1002184.s002.tif]

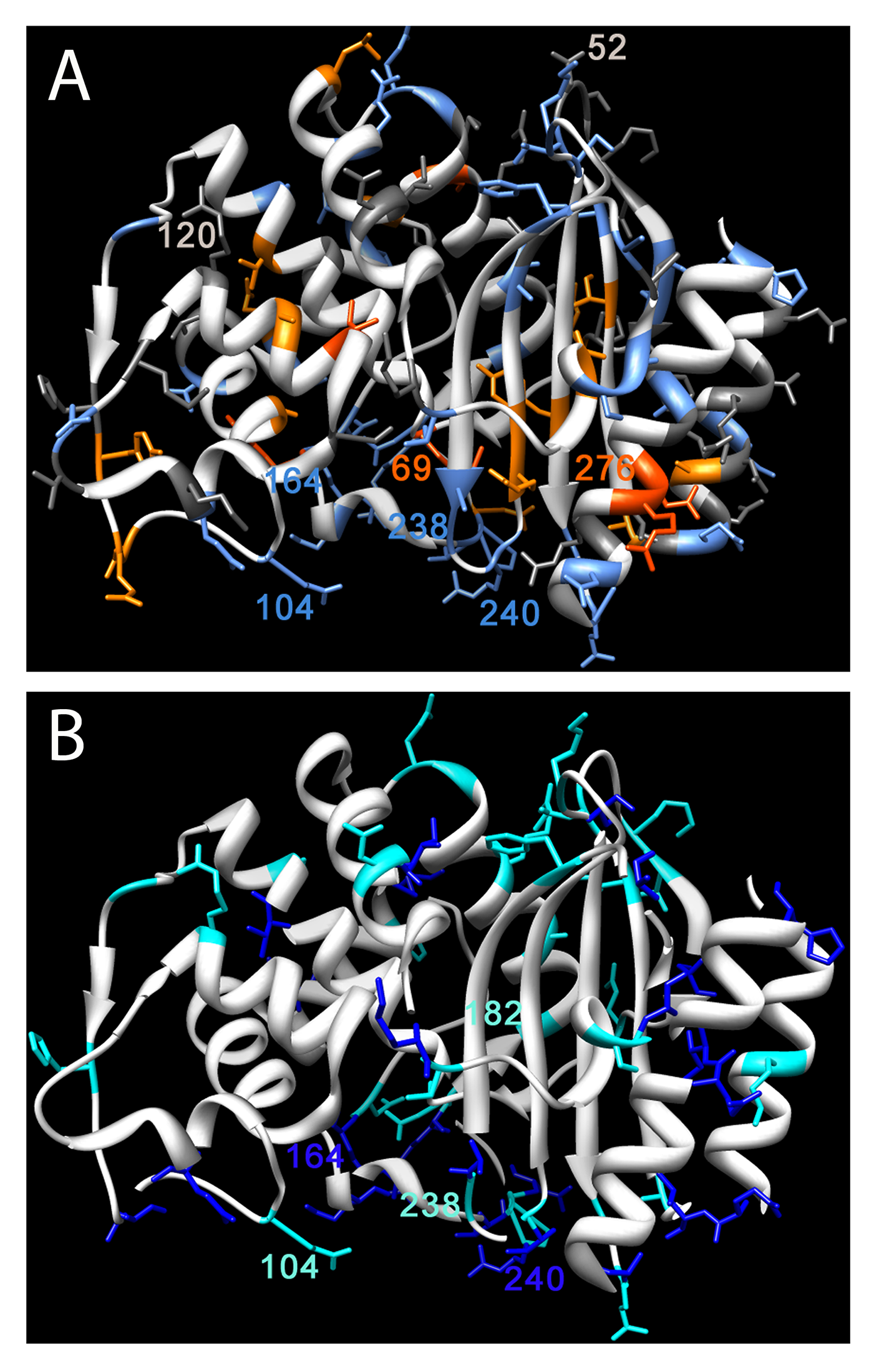

Supplement: Figure S3 — Locations of amino acid residues in the TEM coevolution network and the TEM extended-spectrum community network, mapped onto the TEM tertiary structure (PDB 1ero). (A) Residues in the TEM coevolution network and their three major communities (Figure 1). Residues are colored by community membership: gray (broad-spectrum resistance), blue (extended-spectrum resistance) and orange (inhibitor resistance). The communities do not map to distinct regions of the tertiary structure. Image created with UCSF Chimera [86]. (B) Residues in the TEM extended-spectrum community network and their two major subcommunities (Figure 2). Residues are colored by subcommunity membership: light blue (subcommunity containing the active-site residue 238) and dark blue (subcommunity containing the active site residue 164). The subcommunities do not map to defined regions of the tertiary structure. Image created with UCSF Chimera [86]. (TIF) [file pcbi.1002184.s003.tif]
