# Supplementary material for: Network Models of TEM β-Lactamase Mutations Coevolving under Antibiotic Selection Show Modular Structure and Anticipate Evolutionary Trajectories
Source: PLoS Comput Biol. 2011 Sep 22;7(9):e1002184. doi: 10.1371/journal.pcbi.1002184 (PMC3178621; doi:10.1371/journal.pcbi.1002184)
Supplement: Text S1 — Supporting Methods and Results sections. The Methods section outlines important processing steps for TEM mutations before their inclusion in the network. Key node network centrality metrics are also presented. The Results section highlights the observation that the evolutionary trajectories we predicted by our betweenness centrality ranking are enriched in paths that are parallel to the 104-238-182 evolutionary trajectory. (DOC) [file pcbi.1002184.s013.doc]

**Text S1**

Network models of TEM β-lactamase mutations coevolving under antibiotic selection show modular structure and anticipate evolutionary trajectories

Violeta Beleva Guthrie^1^, Jennifer Allen^2^, Manel Camps^2†^ Rachel Karchin^1∗^

^1^ Department of Biomedical Engineering and Institute for Computational Medicine, Johns Hopkins University, Baltimore, Maryland USA

^2^ Department of Environmental Toxicology, University of California Santa Cruz, Santa Cruz, California USA

^†^ Manel Camps and Rachel Karchin both served as co-mentors for Violeta Beleva Guthrie

^∗^ E-mail: [karchin@jhu.edu](mailto:karchin@jhu.edu)

**Methods**

**Counting of independent co-selection events in reported laboratory evolution experiments**

The directed evolution experiments we included in our sequence database tend to consist of multiple rounds of selection in defined concentrations of β-lactams, so only resistant TEM mutants that are selected in one selection round are used in the next round. In each subsequent round, TEM mutant sequences acquire additional random mutations and only sequences conferring the required level of β-lactam resistance are then selected in the following round, *etc*.

As a conservative way to only include pairs of mutations that arose independently, we did not count the occurrence of a pair of mutations again when it continued to appear through the subsequent selection rounds of a laboratory evolution experiment. In addition, if a pair of mutations was already present in the library of sequences that was used to start the directed evolution experiment, that pair was counted only once, and only if it was present in the first selection round.

**Special case of Q39K**

In the clinical samples database , the Q39K mutation arises independently only once, in the TEM-2 β-lactamase, as demonstrated by earlier studies of the TEM phylogeny . Therefore, naturally occurring TEM mutants have either descended from TEM-1 directly, or through TEM-2. For the construction of the coevolution network, we are interested in how many times a mutation appears and is selected for *independently*, therefore, we removed residue 39 from the alignments of naturally occurring TEMs, but any mutations in this residue found in *in vitro* evolution experiments were included in our model.

**Graph-theoretical node centrality metrics**

**Weighted degree centrality**

The degree of a node is a local measure of this node’s importance in a network. For a weighted network, the node’s degree is the sum of the weights of the links to its adjacent nodes . Degree centrality is defined as node degree divided by the total number of remaining nodes in the network as follows:

Equation S1

**Weighted closeness centrality**

Closeness centrality is a global measure of a node’s importance in a network is defined as:

Equation S2

where the summation in the denominator is over all nodes w in the set ***V*** of all nodes in the network (excepting ***v***) that are reachable from v.

**Weighted betweenness centrality**

Betweenness centrality, an alternate global measure of a node’s importance in a network is defined as follows:

Equation S3

where ***σ_st_ (v)*** is the total number of distinct shortest paths connecting all pairs of network nodes ***(s, t)*** that pass through node ***v***, and ***σ_st_*** is the number of distinct shortest paths connecting node ***s*** to node ***t***. The inequality requirements ensure that only paths that pass through the node of interest ***(v)***, but do not start or end at it, are counted. We developed a simple bidirectional search algorithm, in order to find *all* the distinct shortest paths between a given pair of nodes in a weighted, undirected network. The length of the shortest path was calculated using the weighted distance definition (Equation S2).

**Results**

**Parallel trajectories to 104-238-182 are enriched for high betweenness-centrality**

Amino acid substitutions at 104-238-182 were the most frequent combinationsx obtained from TEM-1 libraries subjected to cefotaxime selection in a recent study . Table S6 lists the reported sequences from this study obtained following cefotaxime selection of TEM-1 and selection of the following libraries of mutants that are negatively epistatic with G238S: R164S, A237T, R164S/G238S, and A237T/G238S. Constituent mutant triplets with nonzero betweenness centrality that appear in these sequences are shown in column 4. Column 5 lists the betweenness-centrality of each mutant triplet and column 6 lists the average betweenness centrality of the triplets in each selection. We split the TEM-1 selection sequences into two groups: sequences that contain the 104-238-182 combination and sequences that do not.

While the TEM-1 descendant sequences (which include a 104-238-182 combination) include only two constituent mutant triplets with nonzero betweenness centrality, trajectories parallel to it are enriched for high-betweenness centrality triplets. We see the most enrichment for these triplets in the selections of libraries that are negatively epistatic with G238S: four out of five in R164S descendants; three out of five in A237T descendants. Strikingly, the triplets that appear in these evolution experiments have high betweenness centrality values, with an average of 19 (R164S) and 31.6 (A237T) per clone. Selection of R164S G238S produced even higher betweeness values (56.2).

Overall, triple mutant trajectories with high positive betweenness centrality appear frequently in trajectories that are parallel to E104K M182T G238S, highlighting the ability of our method to identify paths of special significance for genetic adaptation, although with decreased sensitivity to mutations having a large impact on their own and to global suppressor mutations.

**References**
